# Development of the Impacts of Cycling Tool (ICT): A modelling study and web tool for evaluating health and environmental impacts of cycling uptake

**DOI:** 10.1371/journal.pmed.1002622

**Published:** 2018-07-31

**Authors:** James Woodcock, Ali Abbas, Alvaro Ullrich, Marko Tainio, Robin Lovelace, Thiago H. Sá, Kate Westgate, Anna Goodman

**Affiliations:** 1 UKCRC Centre for Diet and Activity Research, MRC Epidemiology Unit, Cambridge, United Kingdom; 2 Institute for Transport Studies and Leeds Institute for Data Analytics, University of Leeds, Leeds, United Kingdom; 3 Center for Epidemiological Research in Nutrition and Health, University of Sao Paulo, Sao Paulo, Brazil; 4 MRC Epidemiology Unit, Cambridge, United Kingdom; 5 Department for Population Health, London School of Hygiene and Tropical Medicine, London, United Kingdom; Africa Program, UNITED STATES

## Abstract

**Background:**

A modal shift to cycling has the potential to reduce greenhouse gas emissions and provide health co-benefits. Methods, models, and tools are needed to estimate the potential for cycling uptake and communicate to policy makers the range of impacts this would have.

**Methods and findings:**

The Impacts of Cycling Tool (ICT) is an open source model with a web interface for visualising travel patterns and comparing the impacts of different scenarios of cycling uptake. It is currently applied to England. The ICT allows users to visualise individual and trip-level data from the English National Travel Survey (NTS), 2004–2014 sample, 132,000 adults. It models scenarios in which there is an increase in the proportion of the population who cycle regularly, using a distance-based propensity approach to model which trips would be cycled. From this, the model estimates likely impact on travel patterns, health, and greenhouse gas emissions. Estimates of nonoccupational physical activity are generated by fusing the NTS with the English Active People Survey (APS, 2013–2014, 559,515 adults) to create a synthetic population. Under ‘equity’ scenarios, we investigate what would happen if cycling levels increased equally among all age and gender categories, as opposed to in proportion to the profile of current cyclists. Under electric assist bike (pedelecs or ‘e-bike’) scenarios, the probability of cycling longer trips increases, based on the e-bike data from the Netherlands, 2013–2014 Dutch Travel Survey (50,868 adults).Outcomes are presented across domains including transport (trip duration and trips by mode), health (physical activity levels, years of life lost), and car transport–related CO_2_ emissions. Results can be visualised for the whole population and various subpopulations (region, age, gender, and ethnicity). The tool is available at www.pct.bike/ict. If the proportion of the English population who cycle regularly increased from 4.8% to 25%, then there would be notable reductions in car miles and passenger related CO_2_ emissions (2.2%) and health benefits (2.1% reduction in years of life lost due to premature mortality). If the new cyclists had access to e-bikes, then mortality reductions would be similar, while the reduction in car miles and CO_2_ emissions would be larger (2.7%). If take-up of cycling occurred equally by gender and age (under 80 years), then health benefits would be marginally greater (2.2%) but reduction in CO_2_ slightly smaller (1.8%). The study is limited by the quality and comparability of the input data (including reliance on self-report behaviours). As with all modelling studies, many assumptions are required and potentially important pathways excluded (e.g. injury, air pollution, and noise pollution).

**Conclusion:**

This study demonstrates a generalisable approach for using travel survey data to model scenarios of cycling uptake that can be applied to a wide range of settings. The use of individual-level data allows investigation of a wide range of outcomes, and variation across subgroups. Future work should investigate the sensitivity of results to assumptions and omissions, and if this varies across setting.

## Abstract 2

James Woodcock and colleagues present the ICT cycling tool, an open source model for calculating reductions of CO2 emissions and health benefits if cycling replaces car journeys.

## Author summary

**Why was this study done?:**

- Physical activity, such as from regular cycling, is associated with lower rates of mortality and chronic disease.
- Previous studies have modelled the health impacts of people taking up cycling, but the scenarios have tended to be simplistic.
- Policy makers and practitioners need methods and tools to apply to local data to investigate cycling potential and its impacts.

**What did the researchers do and find?:**

- We developed a method to investigate the impacts on health, greenhouse gas emissions, and other outcomes if people were as likely to cycle a trip of a given distance as existing cyclists.
- We applied this method to the English travel survey.
- We developed a web interface to display results for England as a whole, for the nine English regions, and for different groups in the population.
- We found that if the percentage of the English population regularly cycling increased from less than 5% to 25%, there would be approximately a 2% reduction in passenger transport greenhouse gas emissions and years of life lost due to premature mortality.

**What do these findings mean?:**

- The method created can be applied to travel survey data in other countries, and because the code is open source it can be freely improved on by others.
- The study is limited by the assumptions used in the model and by not looking at potentially important health pathways, such as from air pollution and road traffic injuries.

## Introduction

Recent years have seen a rise in cycling-related policies at many institutional levels [1–3]. This interest reflects a recognition that cycling uptake has considerable health, environmental. and transport benefits [4]. Physical activity, such as from regular cycling, is associated with lower rates of mortality and major chronic disease end points [5,6] Road transport produces around 17% of energy related greenhouse gas emissions [7]. Shifting short trips from cars to cycling is a potential health and environmental win-win situation [8,9].

In previous work [10], we showed that if all car trips, using the 7 day English National Travel Survey (NTS) 2010–2012, of fewer than eight miles are switched to cycling, England would achieve a decrease of 19% of passenger car carbon dioxide equivalent (MtCO2e) emissions per year. Such a scenario, in which all shorter car trips are replaced by cycling, is, however, unrealistic and somewhat crude.

This kind of simple scenario is typical of most previous research [9,11] and indicates that we lack models and tools that can provide more flexible and more realistic estimations of which trips could be cycled. This partly reflects the fact that previous studies modelling the impact of increases in cycling (e.g., [12,13]) typically aggregated trip and individual data early on in the process. This limits their potential to develop detailed scenarios or investigate how outcomes vary between different groups. This latter point is important in relation to emerging evidence about inequalities in cycling participation and uptake, with cycling uptake in many contexts being concentrated among relatively affluent, younger, white males [14,15]. Tools are also required that can simultaneously provide estimates of impacts across multiple outcomes, for example, comparing the relative health and environmental benefits of different types of scenarios, while also considering other impacts such as journey time savings. Recent years have seen a rapid rise in the popularity of electric assist bicycles (pedelecs or e-bikes) [16]. Thus, it is important to also estimate how these might impact on health and environmental outcomes. Thus, the Impacts of Cycling Tool (ICT) differs from previous tools (including the Integrated Transport and Health Impact Model [ITHIM]developed by the authors [17] and the WHO Health Economic Appraisal of Transport (HEAT) tool [18]), with its focus on scenario generation and individual-level data.

Therefore, in this study we present a generalisable method for generating cycling uptake scenarios using individual- and trip-level travel survey data. The aim of the study is to describe this method and show selected results for England and English regions.

## Methods

The starting point for our methods is that most existing cyclists do not cycle all their trips (as shown in our analysis, see Table 1 in S2 Text) and are more likely to cycle shorter than longer trips. Based on these findings we modelled what would happen if increasing proportions of the population had the same distance-based propensity to cycle a trip as existing cyclists.

The use of individual-level data enables modelling of multiple outcomes for several population subgroups. The method is applied to the NTS, with supplementary input from other routinely collected surveys. This approach follows calls for data fusion [19]. The results are presented in an interactive web tool that allows users to analyse the original travel survey data and the model results. As we demonstrate, these results can help practitioners and policy makers to quantify the likely transport, health, and environmental impacts of alternative scenarios of cycling uptake.

### Model overview

**Fig 1:**
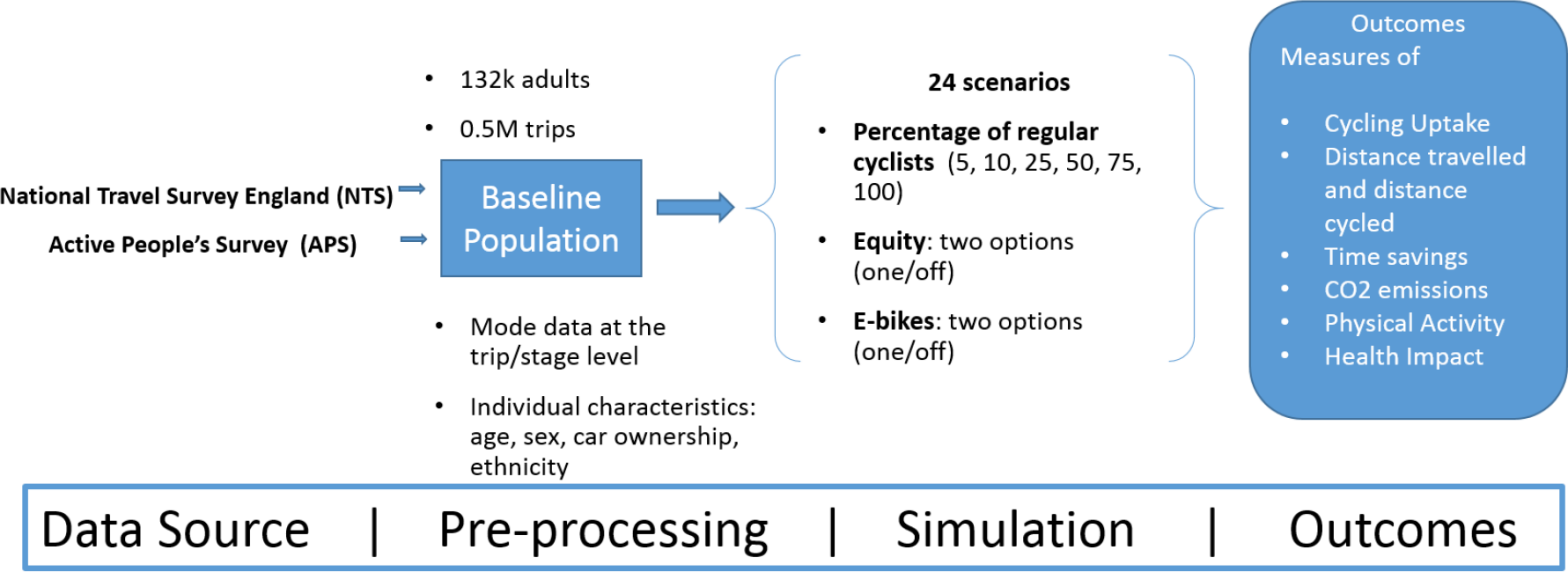
Schematic of main stages in the impacts of cycling tool.

Fig 1 provides a schematic overview of the four main stages involved in creating the ICT: (1) selection of input datasets, (2) baseline population generation, (3) defining and running cycling uptake scenarios, and (4) presentation of the results. This section describes each stage in more detail.

The ICT is created in the programming language R [20], and the code used to generate the model is available online (from http://github.com/ITHIM/ICT-Model), facilitating its use in other countries, regions, or cities.

### Description of data sources

The main data source is the NTS, a nationally representative household survey of travel behaviour in England. The NTS includes one-week travel diary data, with information at both the trip and the stage level (trips are made up of one or more stages and the stages may be of different modes. For example, the main mode of a trip might be by bus, but this might include a short stage of walking to the bus stop, followed by a longer stage of travelling on the bus). From the NTS 2004–2014 sample, we used all 132,000 adults aged 18 years or older in England as the baseline population.

NTS data collection consists of a face-to-face interview and a 7-day self-completed written travel diary [21]. The NTS covers travel by people in all age groups. The interviews were conducted with all household members and gathered information about the household, its individual members, household vehicles, and long-distance journeys that the household members had recently made. Following this, all members of the household completed a week-long travel diary. Data are collected throughout the year. The NTS survey records trips 0.1 miles (160 metres) or longer. However, short walks (<1 mile) are only recorded on the last day of the participant’s survey. The diary is designed such that equal numbers start on each day of the week. To avoid thereby underestimating total walking, short walking trips are replicated six additional times.

NTS data were supplemented with the Active People Survey (APS) (2013–2014) data (559,515 adults). APS was used to provide estimates of physical activity not related to travel.

APS is an annual cross-sectional survey that has been conducted in England every year since 2005, except 2006–2007. The sampling unit comprises all individuals 16 years or older (16+) living in England (using the method described by Rizzo et al. [22]), with no upper age limit. The sample size is a minimum of 500 in most of the local authorities, with some local authorities choosing to boost their sample size. The survey is conducted using a computer-assisted telephone interviewing technique. In every sampled household, only one person aged 16+ is interviewed.

Each round of survey is conducted over a period of one year, starting from October every year. APS reports prevalence of walking and cycling and duration of the activities (number of days and length of time). This information is further classified for utility as well as nonutility purpose. Survey data are weighted to be representative of the 16+ population of each reporting geography.

To inform our scenarios involving e-bikes, we also used the NTS with the 2013–2014 Dutch Travel Survey. Unlike the NTS in England, this survey records e-bikes as a separate mode. Like the NTS in England, the Netherlands survey includes all-age categories. Unlike in England, the sampling unit is individuals, not households. It uses a mixture of approach methods, starting with internet, then telephone, then face to face. The Dutch Travel Survey records all trips 100 metres or longer.

### Generating the baseline population

#### Baseline travel behaviour

The baseline population was generated from the individual-level questionnaire data plus trip-level travel diary data collected in the NTS. The travel diary records every trip (except short walks) in the past week made by each individual. From the many variables available in creating the ICT, we use five key individual-level characteristics (age, gender, ethnicity, socioeconomic group, and car access) and three trip-level characteristics (main mode, duration, and distance). For public transport trips, the model also uses a fourth key variable, duration of time spent walking (derived from stage-level data. This captured 97% of all minutes reported in NTS as having been spent in active travel—walking or cycling—as part of a trip for which the main mode was motorised).

For socioeconomic group we used the British socioeconomic classification, National Statistics Socio-economic classification (NS-SEC), which is based on occupational type.

#### Baseline physical activity

Travel-related physical activity is derived from the NTS data based on minutes spent walking and cycling, as well as their intensity.

Metabolic Equivalent Tasks (METs) are a method for combining time spent in activities of different physical intensity, with higher MET values being allocated to more intensive activities [23]. In our model, we used the extended concept of marginal METS, which only considers the marginal activity over and above rest [24]. The marginal MET intensity multiplied by the duration (intensity × duration) gives the marginal MET hours (MMEThs) for the activity (walking for transport = 2.6 MMETs [25]; cycling on a pedal bike for transport = 4.6 MMETs [25]; cycling on an e-bike = 3.5 MMETs [26]). Individual weekly travel-related physical activity was obtained by summing the MMEThs over all of each individual’s cycling trips, walking trips, and walking stages as part of public transport in the NTS. In Supporting information (S1 Text), we explain how we calculated an individual's total weekly physical activity by additionally estimating their non-travel physical activity. Non-travel physical activity was assumed to remain unchanged in all scenarios [27].

### Generating the scenarios

To generate new cycling trips, we applied a two-step process:

1. Step 1: an individual becomes a regular cyclist. Each non-cyclist is assigned a probability of becoming a cyclist. Some individuals are probabilistically converted from being a non-cyclist to being a regular cyclist.
2. Step 2: a trip is switched to cycling. Among individuals who become regular cyclists, each trip is assigned a probability of being cycled based on the observed propensity to cycle a trip of that length observed among existing cyclists (e.g., a 10 mile trip has a lower probability of being cycled than a 2 mile one; see Table 2 in S2 Text).

This two-step method mimics real life situations more realistically than a one-stage process of applying the probability of switching to cycling independently to each trip, as it produces a more realistic clustering of cycling trips at the individual level. Both steps are described below in more detail.

#### Step 1: Modelling non-cyclists becoming regular cyclists

Among adult participants in England in the 2004–2014 NTS, 4.9% were ‘current cyclists’, defined as those who reported at least one cycle trip in the past week in their travel diary. This proportion varied by age and gender: 8.4% for males aged 18–59; 3.7% for females aged 18–59; 4.4% among males aged 60–79; and 1.6% among females aged 60–79.

For a given scenario, we calculate the difference between the baseline percentage of the population who are regular cyclists and the scenario percentage; e.g., for 25% scenario for England, the increase is 25%–4.8% = 20.2%.

In an equity scenario, any non-cyclist has the same probability of becoming a regular cyclist, regardless of age and gender. Hence, the additional 20.2% of the population are selected at random from those without cycling trips at baseline. In non-equity scenarios, the probability of a non-cyclist becoming a regular cyclist is in line with the current age and gender differences observed among current cyclists. Hence, a man aged 18–59 who does not cycle at baseline would be nearly twice as likely to become a cyclist as an older man.

#### Step 2: Modelling mode switch among regular cyclists

For regular cyclists, a probability function was applied to all their trips that determined how likely each trip was to be cycled, given the distance band of the trip.

In both equity and non-equity traditional bike (non–e-bike) scenarios, we assumed that age and gender differences in cycling speed and willingness to cycle longer trips were maintained using data from NTS 2004–2014; e.g., older cyclists would continue to cycle slower than younger cyclists and be disproportionately deterred by longer distances. In the Supporting information, we have described this approach in more detail (see S2 Text).

In e-bike scenarios, we assumed that all new cyclists had access to e-bikes. Based on analysis of the Dutch National Travel Survey 2013–2014, we found that 53% of the e-bike owners also owned a traditional bike and often used this for shorter trips. We used the Dutch survey to estimate both the likelihood of an e-bike owner using an e-bike as opposed to a traditional bike and the distance-based cycling propensity (see Table 3 in S2 Text). For these e-bikes trips, cycling speed and the probability of cycling longer trips were assumed not to vary by age and gender in both equity and non-equity scenarios (see Table 4 in S2 Text).

It should be noted that someone may become a regular cyclist but not have any trips allocated to cycling for that specific week, because of the combination of the trips they are making and chance. Thus, after Step 2 the population can be split into four groups:

1. Existing cyclist: an individual who reports trips made by cycle in their travel diary and so is a cyclist in both the baseline population and in the scenario.
2. Regular cyclist: an individual who is not a cyclist in the baseline population but who is modelled as becoming a cyclist in the scenario (but may or may not actually switch any trip to cycling).
3. New cyclist: an individual who is not a cyclist in the baseline population, who is modelled as becoming a cyclist in the scenario, and who switches at least one of their trips to cycling. New cyclists are thus a subset of regular cyclists.
4. Non-cyclist: an individual who is not a cyclist in the baseline population and who is not modelled as becoming a regular cyclist in the scenario.

#### Combining scenario elements to generate up to 24 separate scenarios per region

In summary, in Step 1 an increasing proportion of the population become regular cyclists, either with or without an assumption of age and gender equity in uptake. In Step 2 an ‘e-bike’ parameter is applied in determining which trips are switched to cycling and e-biking. These three parameters used to generate the ICT scenarios are summarised in Table 1.

**Table 1:** Three core parameters used to create impacts of cycling tool scenarios.

Concept	Application	Values	Meaning
Percentage of the population who are regular cyclists	Step 1	[5%]^†^, 10%, 25%, 50%, 75%, 100%	Determines the percentage of the population considered for having trips switched to cycling
Equity	Step 1	0 (no) versus 1 (yes)	Assumes equal likelihood to become a regular cyclist across age groups and genders
E-bike use	Step 2	0 (no) versus 1 (yes)	Assumes all new cyclists have access to e-bikes and use them for some of their cycling trips, particularly the longer ones

^†^5% scenario was only run if the baseline proportion of cyclists in a region was below 4%.

The scenarios are specified for England as a whole and for each of the nine English regions (North East, North West, Yorkshire and the Humber, East Midlands, West Midlands, East of England, London, South East, and South West). For example, the set of values “South West, proportion of population who are regular cyclists = 10%, equity = 1, e-bike = 0” would describe a scenario with 3.1% additional regular cyclists (10%- observed baseline value of 6.1% cyclists in the South West) + men/women/young/older adults having the same probability of becoming regular cyclists as each other and no use of e-bikes.

The three parameters in Table 1 produce a total of 24 potential scenarios for each region: 6 (% of population who are regular cyclists) * 2 (equity on/off) * 2 (e-bikes on/off). However, in practice, once 100% of the population are regular cyclists, then the equity setting for becoming a regular cyclist is irrelevant. In addition, in England as a whole and in five of the nine regions, the baseline proportion of cycling was close to or greater than 5%, and hence the four 5% scenarios were not included. In total, therefore, the number of scenarios modelled was 6*19 + 4*23 = 206.

### Outcomes

The outcomes calculated in the ICT are grouped under the following headings:

- Cycling uptake, miles cycled, mode share: including the number of new cyclists in the population, the total distance travelled by bicycle, and the cycle mode share (defined as the proportion of transport trips made by cycle).
- Journey times: trips switched to cycling are recalculated for duration, using average cycling speeds (see Table 3 in S2 Text) and self-report distance. Speeds for traditional bikes were calculated from the NTS, and for e-bikes from a Dutch report. Trip duration is necessary to calculate physical activity energy expenditure for trips switched to pedal bikes or e-bikes. Comparing the new calculated trip duration against the previous self-reported duration allows estimation of the change in travel time for each trip and thus what proportion of trips become faster or slower, and by how much, when switched.
- Car miles and CO_2_ emissions: the model calculates total distance travelled by car and the reduction in car miles relative to baseline. Indicative results for CO_2_ are estimated using an average value per private motor vehicle of 0.313 kg CO_2_ equivalent per mile [10,28]. Reductions in public transport trips have not been included because a decrease in passenger numbers may not directly lead to a reduction in distance travelled by the bus or train fleet.
- Physical activity: in the scenarios, physical activity changes because of increases in time spent cycling and e-biking and reduction in time spent walking, including walking for public transport. We present results both in terms of mean MMETh per week and, derived from this, as the proportion of the population achieving physical activity recommended levels. The World Health Organization (2010) recommends at least 2.5 hours per week of moderate intensity activity, which we interpreted as equivalent to 8.75 MMETh per week, based on the midpoint of the moderate activity range (3.5 MMETs). The World Health Organization further recommends [29] that people should aim for a higher volume (5 hours/week), equating to 17.5 MMETh per week.
- Health: the health impact of cycling is calculated using a Comparative Risk Assessment, with individual-level exposure estimates. We estimated the change in risk of all-cause mortality for a change in physical activity for each individual using a large pooled meta-analysis of cohort studies [30]. The risk function is nonlinear and the baseline activity levels provide the starting point for each individual on that nonlinear curve. The change in risks is applied to regionally specific data for England from the Global Burden of Disease Study for each gender and age group [31]. Impacts were modelled as compared against a hypothetical constant state without an explicit time dimension or time lag. Detailed methodology is explained in the Supporting information section (see S3 Text).

## Results

### Illustrative results from the model

**Fig 2:**
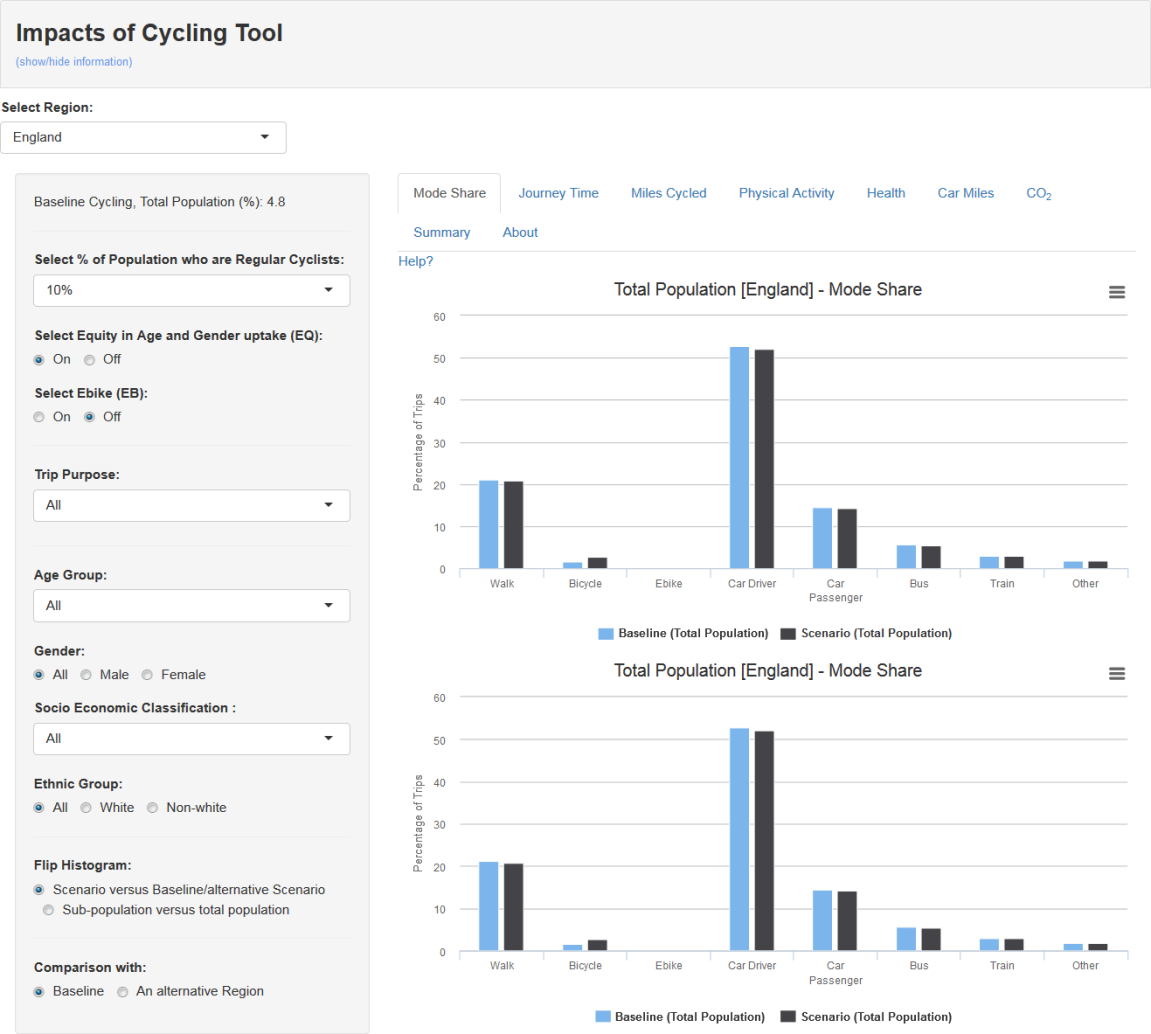
Impacts of cycling tool online interface.

The large number of scenarios generated and their multiple subgroups mean that in this section we can only present selected, illustrative results. We have chosen to focus on the results for the scenarios in which 25% of the population are regular cyclists, without age and gender equity or e-bikes (‘exemplar scenario’). We encourage the reader to use the web tool at https://pct.bike/ict to investigate other results. Please see Fig 2 for the landing page of the interactive interface.

### Baseline data visualisation

**Fig 3:**
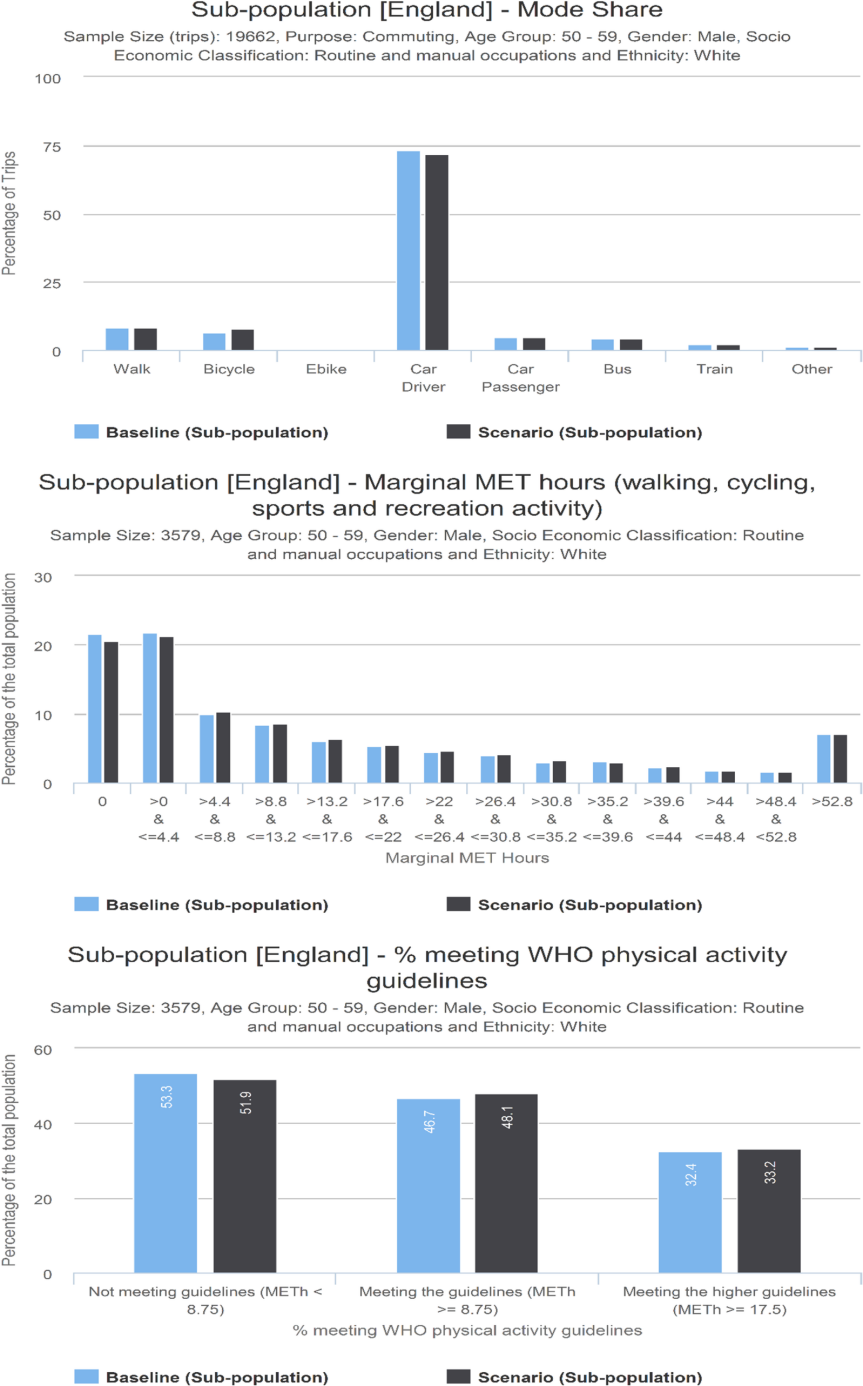
**Screenshot of interface showing (a) Mode share and (b) physical activity levels for whole population and selected subgroup. (c) Percentage of whole population and selected subgroup meeting the WHO physical activity guidelines (MMETh > 8.75 and MMETh > 17.5).** MET, Metabolic Equivalent Task; MMETh, marginal MET hour.

The functionality of the web tool can be illustrated by starting with the baseline data from the NTS and fused data from the APS. Fig 3A shows the modal share (based on main mode) for one trip purpose (commuting trips), for the whole population, and for a specific subgroup (white men aged 50–59 in routine occupations). Similarly, Fig 3B shows the population distribution of physical activity volumes (MMETh per week) in the whole population and the selected subgroup. The tool also shows the percentage of the population meeting WHO physical activity guidelines (Fig 3C).

In the interface, the actual values can be seen by hovering over the bars. The data can be stratified according to age group, gender, socioeconomic group, and ethnic group (white versus non-white). More specific ethnic groups were not used as sample size would be too small for some regions. The phrasing white/non-white is used as it was the one reported in the survey and the broad grouping provided a sufficient sample for all regions. For more information, see S1 and S2 Tables).

### Number of cyclists and miles cycled

**Fig 4:**
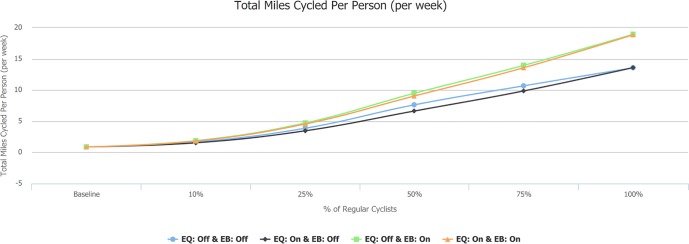
Miles cycled per person per week. EB, e-bikes scenario; EQ, equity scenario.

The values for the number of people who cycle at least one trip per week increase in line but are slightly slower than the proportion of the population who are regular cyclists (see S4 Fig). E-bikes tended to marginally increase the total number of cyclists (as e-bikes increase the number of trips able to be cycled). E-bikes unsurprisingly produced much higher distances cycled (Fig 4). Total distance cycled was slightly higher with unequitable take-up, particularly in non–e-bike scenarios, as younger men are more likely to cycle longer trips. In the scenarios with 25% regular cyclists, the mean distance cycled increased to just under 3.5 miles per person per week.

At baseline the lowest and the highest percentages of the population cycling were the North East, with 3.1% (0.6 miles per person), and South West, with 6.1% (1.1 miles per person). In the exemplar scenario, cycling was similar in the two regions (22.5% of the population, 4.2 miles in the North East, and 23.3% of the population, 4.1 miles in the South West).

### Mode share

The interface allows visualisation of the trip mode share for any of the scenarios, and to compare these with the baseline.

**Fig 5:**
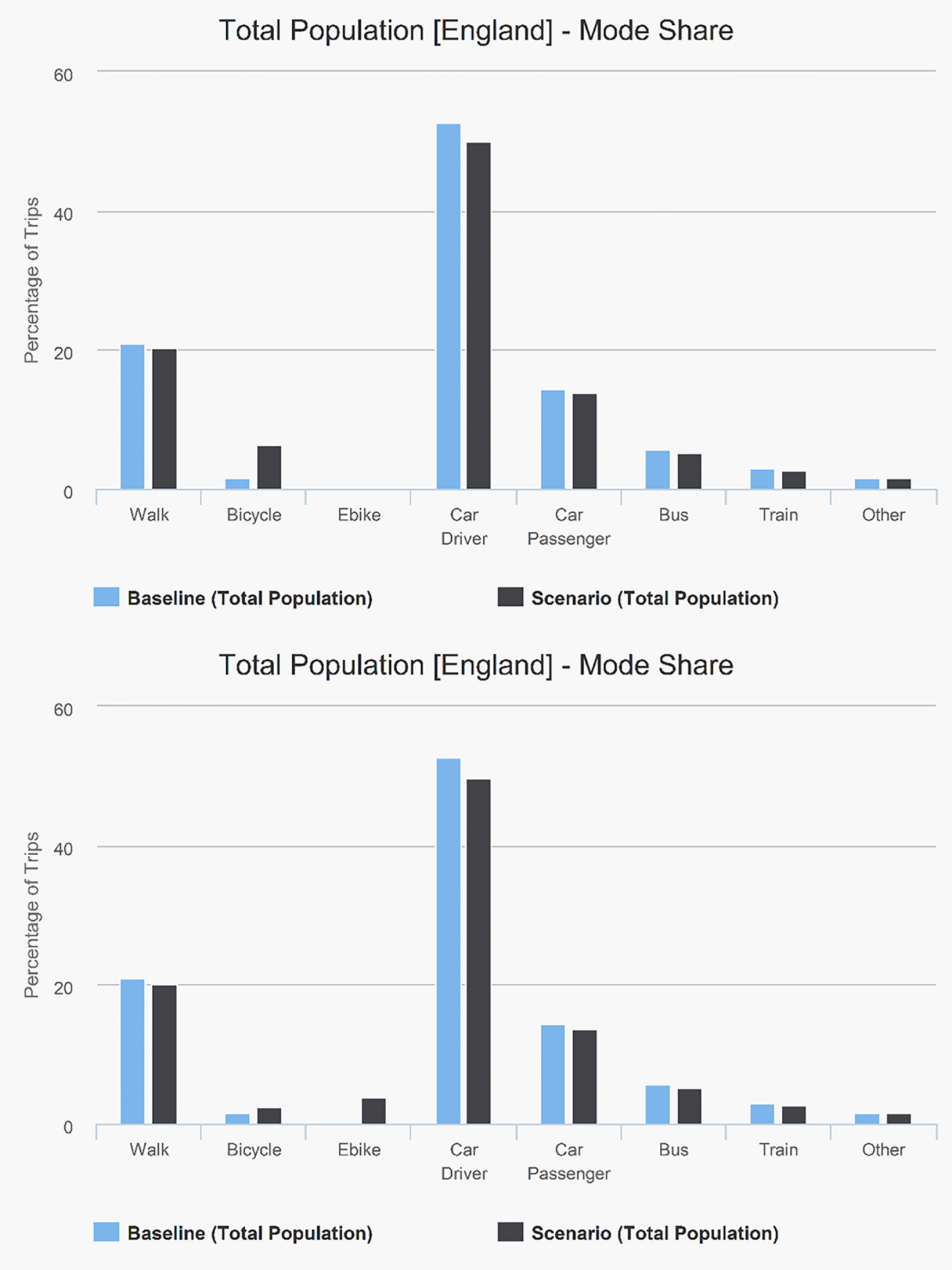
Mode share baseline compared with scenario 25%, equity off, e-bike on/off.

Fig 5 shows the trip mode share for the exemplar scenario (see S3 Fig for a comparable graph with e-bikes on). The overall mode share for traditional bicycles reaches 6.3%, while for cars (driver + passenger) it falls from 67.1% to 63.8%. In the non-white population, the car mode share (52.4%) and cycling mode share (0.9%) are much lower at baseline than for the white population. For the same scenario in the non-white population, the car mode share falls to 49.0% and cycling increases to 6.8%.

### Change in physical activity

**Fig 6:**
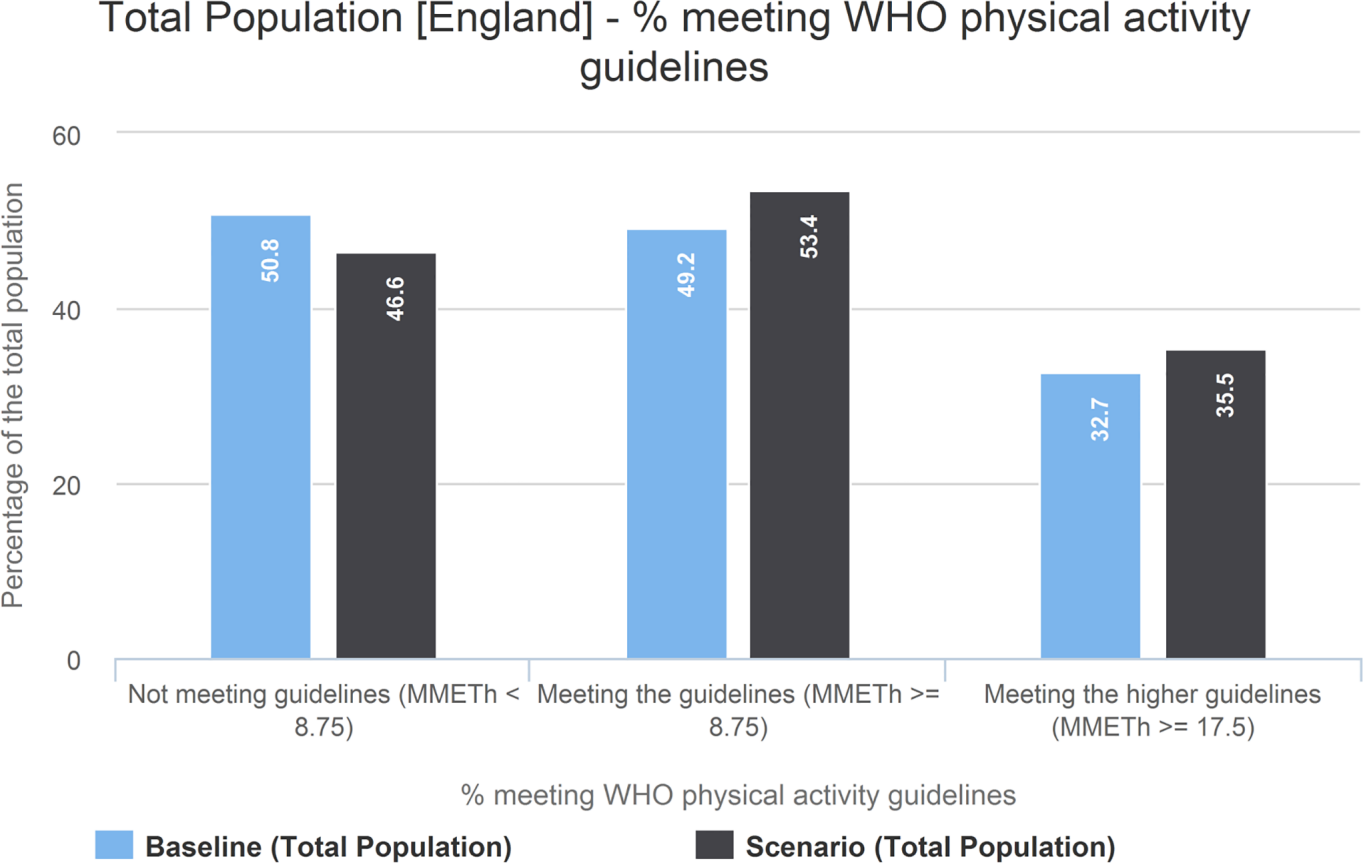
Percentage of the population achieving minimum recommended physical activity per week. Exemplar scenario with 25% regular cyclists, equity off, e-bikes off.

As the percentage of regular cyclists increased, mean marginal METs per person per week for the population increased from 3.5 to 4.5 (see S6 Fig) in in the exemplar scenario. This corresponded to just over a 4% increase in the proportion of the population meeting minimum physical activity guidelines (at least 8.75 MMET hours per week; see Fig 6). Similarly, the proportion of people who achieve the higher recommended activity level (17.5 MMET hours per week) increased from 32.7% to 35.5%. For a given percentage of the population being cyclists, increases in mean physical activity were marginally smaller in scenarios with equitable take-up and e-bikes.

The West Midlands started with the lowest percentage of the population at baseline achieving the lower recommended physical activity levels (45.4%), increasing to 50.6% in the exemplar scenario. Nationally, physical activity levels were lower among the non-white population at baseline (45.1%), increasing to 50% in the exemplar scenario.

### Health outcomes

**Fig 7:**
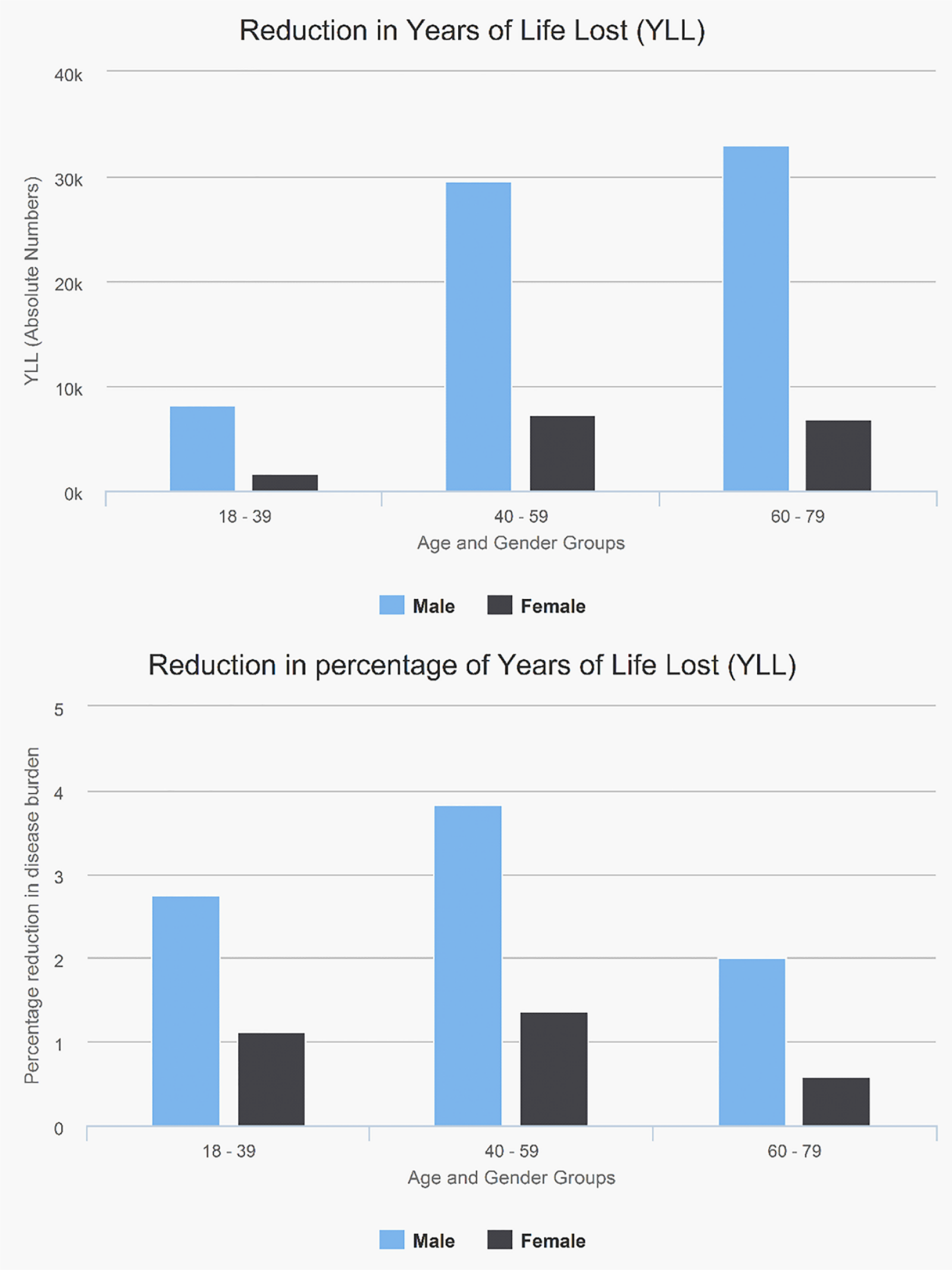
**(a and b) Absolute and percentage reduction in burden on premature mortality (YLLs) for England, exemplar scenario with 25% regular cyclists, equity off, e-bikes off.** YLL, year of life lost.

The increase in physical activity translates into a reduction in the disease burden from physical inactivity. In the exemplar scenario, the increase in cycling would avert 2.1% of premature mortality for the population aged 20–79 years. Because existing gender inequalities in cycling were assumed to be perpetuated in this scenario, benefits were larger among men than women (e.g., among people aged 40–59, 5.1% in males, 1.7% in females; see Fig 7). Because existing age inequalities in cycling were assumed to be perpetuated in this scenario, relative benefits were greater in the middle-aged compared with older adults. However, even with lower uptake, absolute benefits were marginally greater in older adults (see Fig 7), because of higher absolute mortality rates at older ages.

Unlike physical activity, health benefits for a given percentage of the population being cyclists were greater in scenarios with equitable versus non-equitable uptake. E-bikes were associated with greater benefits in equity but not non-equity scenarios. If applied to the entire English population for the exemplar scenario (25% of the population being regular cyclists without equity), the benefits would be about 95,000 years of life lost (YLLs) without e-bikes. In the equivalent e-bike scenario, approximately 93,000 YLLs would be saved. With equity, this would be about 100,00 without e-bikes and 105,000 with e-bikes.

**Fig 8:**
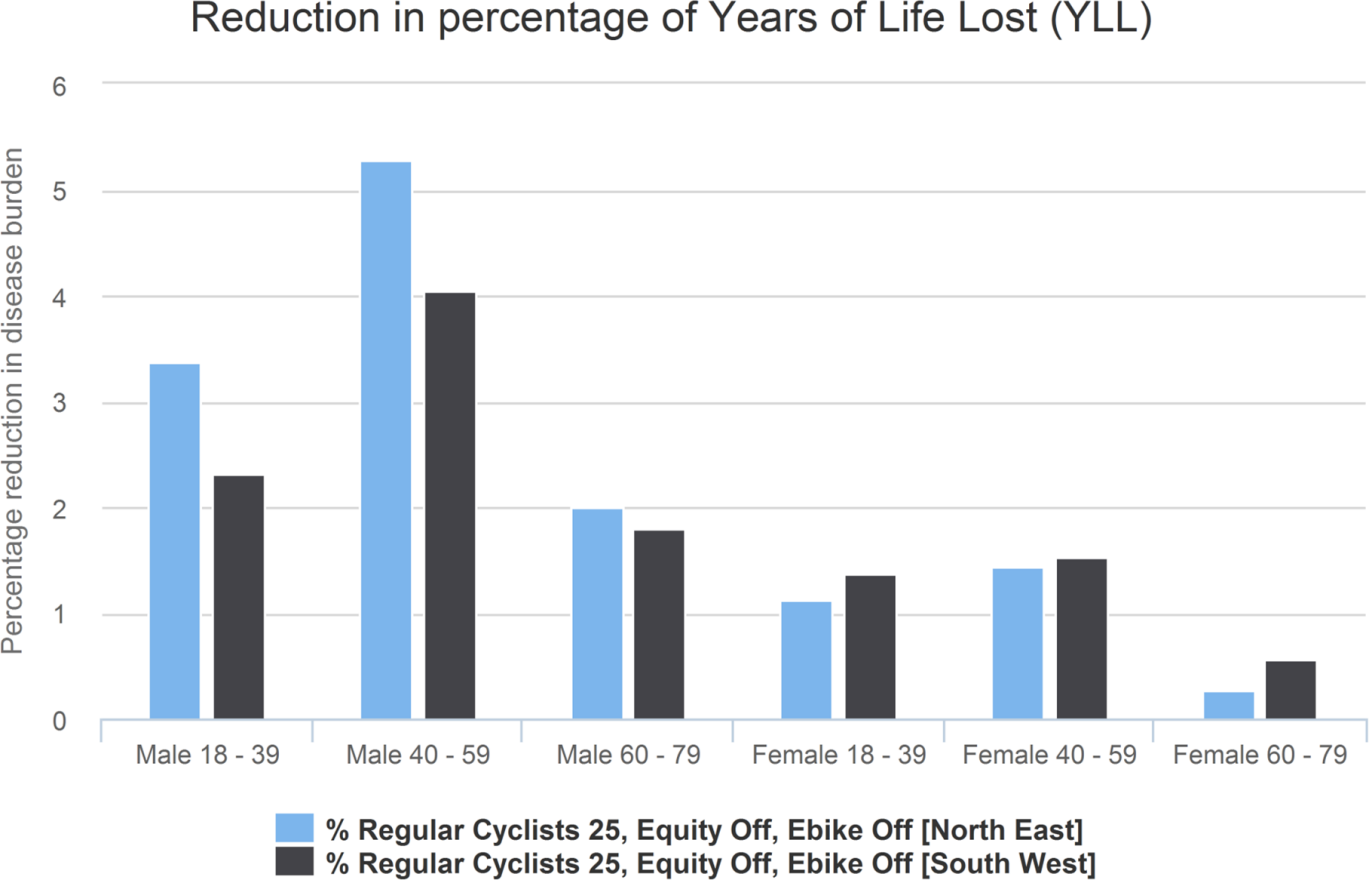
Reduction in percentage of number of deaths. YLL, year of life lost.

In Fig 8, we display results for the exemplar cycling scenario for the North East and South West regions. Health outcomes are broken down by age and gender groups. It shows that the North East would gain more benefits for men but fewer for women, compared with the South West.

### Change in car miles/CO_2_ emissions

**Fig 9:**
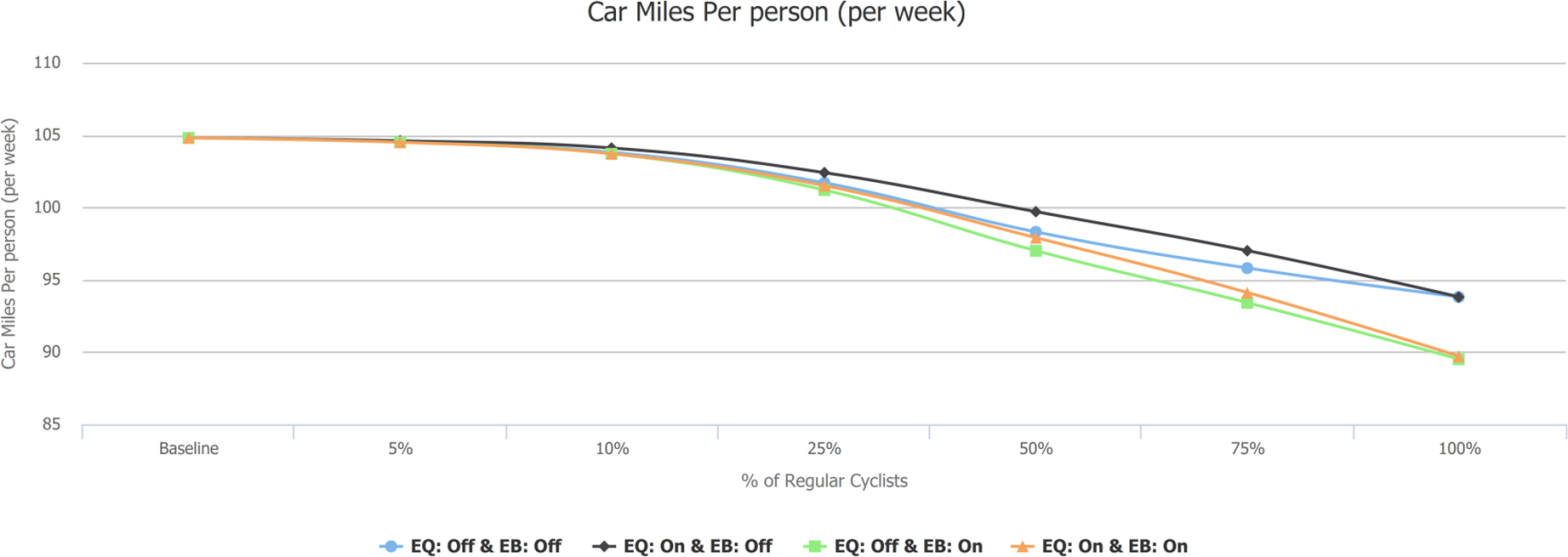
Car miles per person per week. EB, e-bikes scenario; EQ, equity scenario.

In the exemplar scenario (see Fig 9), average distance by car in England as a whole falls by 2.2% (2.5 miles per person per week). In the equivalent scenario with e-bikes, the distance by car is reduced by 2.8% (3.2 miles per person per week). Because CO_**2**_ is modelled as a simple multiple of car miles, proportional changes in CO_**2**_ were the same as for car miles (see S5 Fig).

In terms of regional variation for the exemplar scenario, we find a larger absolute but smaller relative fall in car miles per person, comparing the high-driving South West (1.9%, from 135.4 to 132.8) with low-driving London (2.5%, from 59.5 to 58).

### Journey time

Unsurprisingly, the majority of trips switched from car to bike were slower, while all trips switched from walking to bike were faster. For public transport (including waiting times), more than twice as many trips would be faster.

For the population as a whole in the exemplar cycling scenario, just under 57% of the trips switched would be slower by bike. We found, however, large differences by ethnicity and gender. Among women, just over 50% of trips would be faster by bike. Among non-white women, over two thirds of trips would be faster by bike. E-bikes made a relatively small difference to these findings, as, although they are faster, some of the trips cycled would be longer.

In the exemplar scenario, the West Midlands (with lower levels of walking), around 62% of trips would be slower, while in London, with high walking and public transport, a large majority (66%) of trips would be faster.

## Discussion

### Summary of principal findings

The study has demonstrated that if non-cyclists were as likely to cycle short- to medium-distance trips as existing cyclists, there would be considerable benefits in terms of both health and greenhouse gas emissions from transport.

In terms of physical activity in our exemplar scenario (25% of the population regular cyclists without equity or e-bikes), there would be a 1 marginal MET hour increase per week in mean energy expenditure. As a result of this, the health burden for England’s population would be reduced by around 100,000 YLLs in a single accounting year (2.1% of total burden among those under 80 years). Previous work has demonstrated the importance of cycling for meeting physical activity targets in the high-cycling Netherlands [32]. If 100% of the English population became regular cyclists, then the mode share would reach 23%, very close to the Netherlands.

In our exemplar scenario, cycling would replace 2.2% of car miles, and the mode share for car drivers would fall from 52.7% to 49.9%. If we assumed that the increase in cycling were accompanied by widespread use of e-bikes, then we would see a notably greater replacement of car miles (2.8%) and a higher cycling mode share (6.5% [2.6% traditional bikes, 3.9% e-bikes] versus 6.3% with traditional bikes). Health impacts would be marginally smaller—95,000 versus 93,000 YLLs.

We have also demonstrated how outcomes vary by subpopulation and region, including the substantial journey time savings in London and for non-white women.

### Implications for policy

The code underlying the ICT is open source, enabling anyone to explore the assumptions underlying the estimates of cycling uptake. Crucially, from a policy perspective, the results can be freely accessed online by policy- makers, practitioners, and the public to investigate how different uptakes of cycling would impact across a range of outcomes.

Unsurprisingly, our results indicate that the trips that were most often faster when cycled were walking trips (although many public transport trips and even some car trips were also faster by bike). There is a tension here—cycling for transport is usually promoted to improve health and sometimes reduce greenhouse gas emissions. Replacing walking trips by cycling does not increase physical activity, and, moreover, walking and cycling are often seen as joint goals. However, the big goal of much transport investment is to reduce travel times, and here cycling has a clear advantage over walking. Although this goal has arguably been overvalued in the past [33], it is an important aspect of individuals’ transport preferences [34]. There is also the issue of transport equity; cycling may reduce travel times for those without car access. Beyond this, there are other aspects of walking and cycling that cannot be captured in this study. For example, in a city that is scaled to the car, the environment for walking is likely to be sensorily impoverished. However, in a sensorily richer environment, walking allows more interactions and a finer-grained appreciation of the environment.

### Strengths, weaknesses, and directions for future work

Previous studies have modelled the health impact on a mode shift to active travel in England and other settings [9,11,35,36]. The approach in the ICT has advantages over this previous work. Firstly, it uses locally relevant (English) data to derive propensities for cycling trips of different distances (only using Dutch data for e-bikes) and, secondly, it applies these to individual trips. The application to individual trips makes it much clearer which trips would be cycled under different assumptions and thus allows calculation of a wider range of outcomes and analysis of results for subgroups. Thirdly, this work is created using open source software and comes with a web tool that allows detailed interrogation of the results. An interesting avenue for further work would be to make the results of this model applicable to localised estimates of cycling uptake, such as those generated by the Propensity to Cycle Tool [37]. Below, we outline some of the limitations of the current model and ways that these could be overcome.

The great strength of individual-level modelling is that population heterogeneity can be represented. In this version of the ICT we have been able to follow a few lines of inquiry, e.g., examining impacts by age, gender, socio economic status, and a simply dichotomised ethnicity. Given debates on sustainable transport and gentrification, this is pertinent [38,39]. We anticipate in the future calculating propensities to cycle stratified by ethnicity and car ownership. Given the strongly divergent finding by ethnicity, future work should investigate further, including how impacts vary between ethnic minority populations in England. In other settings, different stratifications may be more relevant.

The uptake of cycling is modelled in a simple manner. An alternative would be a predictive model including variables such as climate, time of day, and travelling accompanied. We did not pursue this, as we suggest it would be less clear to model users and less generalisable, with the modifying effect of such factors likely to vary by setting.

Our scenarios assume that current trip numbers and trip distances remain unchanged. We may be missing suppressed travel demand, particularly among people without car access, who may experience greater time savings from switching to cycling. This is supported by recent empirical evidence reporting higher use of new infrastructure in households without car access [40]. We assumed that people were equally likely (based on distance) to switch from any other mode to cycling. If time saving is a dominant factor in individual decision-making (as opposed, e.g., to health benefits or cost savings), then many more trips would be expected to switch from walking, thereby reducing the health and greenhouse gas emission gains.

Our study relies on self-reported data, and the limitations of this are well established. Trip diary data have been shown to be reasonably accurate on average, e.g. [41], but more work needs to be done on larger populations to investigate potential differential biases by mode, trip purpose, age, and gender.

Our model likewise assumes that the mean speed of each mode is both accurate and remains unchanged in our scenarios. The model thus does not consider how an increase in cycling, as one of the most space-efficient modes of transport, could reduce congestion and so potentially speed up traffic for other modes. To do this well would require detailed local analysis, but some indication of congestion benefits is provided by the fact that almost 70% of new cycle trips were previously made by car. To the extent that congestion does decrease, this could be incorporated within estimates of CO_2_ emissions. As cycling would tend to replace shorter car trips in more congested urban settings, our current approach is likely to underestimate the total emission savings.

We assume in the traditional bike scenarios that new cyclists have the same distance-based propensities as current cyclists. This may underestimate the potential, as current cyclists may not cycle all the trips they would like; e.g., if only some trips have adequate infrastructure. Alternatively, one could argue that this overestimates the potential, as new cyclists may not have the same distance-based propensities as current cyclists (lower fitness, less knowledge about quiet and safe routes, etc.). As evidence becomes available on how cyclists behave when cycling increases [42], we will be better able to parametrise near-term scenarios.

#### Barriers to cycling

Fear of motor traffic is probably the main barrier to cycling in most contexts [43], with high rates of near misses reported [44]. However, assuming this is overcome by improvements to infrastructure [45,46] and driver behaviour, other barriers (beyond distance) would remain for specific trips. Carrying passengers (e.g., children) and heavier or bulkier items can be difficult with traditional bikes, although electric assist cargo bikes can help. Hilliness likewise reduces propensity to cycle, and if investigating variation in propensity at smaller geographies, then inclusion of hilliness would be an important variable to include [47]. As the model is based on individual data, future work can relatively easily include other variables in the original survey. However, without more spatially detailed data (such as in the Propensity to Cycle Tool [37]), inclusion of hilliness is difficult.

#### Health benefits and harms

The use of individual-level data allows a more nuanced calculation of the population impact fraction compared with approaches using aggregate data (e.g., [36]). By using data fusion to estimate total nonoccupational activity, we are able to model in a realistic way with nonlinear dose response relations [48,49].

Limitations relate to both the choice of health pathways and health outcomes. Benefits may be over- or underestimated because we are not modelling injury risks and air pollution exposure. Earlier studies have found that benefits from cycling at the population level substantially exceed the harms from injuries for most population groups [9,11]. It should also be noted that although injury risks are typically higher amongst cyclists (potentially, injury risks for e-bike users might be higher because of their greater speed, although recent research as part of the Near Miss project found fewer near misses for faster cyclists [44]) than among motorised road users [50,51], fewer cars on the roads would reduce population level risks. An earlier modelling study for England found that gains from physical activity were far larger than were changes in injury, and that in some active travel scenarios, total injury burden fell [35]. However, for younger people, with lower short-term risk of noncommunicable disease, the short-term health benefits may be less clear [13]. Equally for air pollution, cyclists would typically inhale more pollutants, but the emissions would fall. The marginal impact of additional air pollution when cycling with English levels of air pollution is considerably less than benefits from physical activity [52].

By focusing on premature mortality, we are missing the benefits that might also be achieved by reducing morbidity [24]. However, earlier studies have found that estimates of impact using relative risks for all-cause mortality may be substantially higher than when estimating through individual diseases [35]; thus, the exclusion of additional benefits from morbidity may help to reduce the risk of overestimation.

#### Uncertainty and sensitivity analysis

The modelling is probabilistic in terms of the matching, becoming a cyclist, and switching trips. We have not, however, implemented Monte Carlo analysis to investigate the uncertainty of our results. Future work should investigate the sensitivity of the model to assumptions, including testing the Value of Information, to guide future model development [53].

#### Extension to other settings

The ICT continues to evolve. We invite other researchers to improve and expand the approach using our open source code, which is available at https://github.com/ITHIM/ICT-Model, and welcome enquiries about collaboration on its development and application. The methods can be applied to any individual-level travel survey data, as are available in many cities, regions, and countries around the world. The approach could also be applied to synthetic data from the activity-based travel model (e.g., [54]). Comparing results across different settings will facilitate understanding of the reasons for differences in cycling levels and the extent to which cycling can help tackle the societal challenges of inactivity and greenhouse gas emissions.

## Supporting information

S1 Text Calculating the total weekly physical activity of individuals. (DOCX)

S2 Text Probability of cycling a trip. (DOCX)

S3 Text Further details of health calculations. (DOCX)

S4 Text Further tables (mode share for all four possible combinations of 25% scenario, and YLL reductions for males and females, for all four possible combinations of 25% scenario). YLL, year of life lost.
(DOCX)

S1 Table Active People's Survey's ethnicity table. (XLSX)

S2 Table Description: National Travel Survey (England)'s table on individual ethnicity. (XLSX)

S1 Fig Probability of cycling a trip of a given distance among adult English cyclists in the National Travel Survey and among adult Dutch e-bike owners. (PNG)

S2 Fig All-cause mortality—Total population. (PNG)

S3 Fig Mode share baseline compared with e-bike scenario. (PNG)

S4 Fig Percentage cyclists in the total population. (PNG)

S5 Fig Percentage reduction in CO_2_ from private personal transport. (PNG)

S6 Fig Marginal METs per person. EB, e-bikes scenario; EQ, equity scenario; MET, Metabolic Equivalent Task.
(PNG)

S7 Fig Faster and slower trips. Scenario regular cyclists 100%, equity on, e-bikes off.
(PNG)

## Abbreviations

APS: Active People Survey
ICT: Impacts of Cycling Tool
ITHIM: Integrated Transport and Health Impact Model
MET: Metabolic Equivalent Task
MMETh: marginal MET hours
MtCO2e: passenger car carbon dioxide equivalent
NS-SEC: National Statistics Socio-economic classification
NTS: English National Travel Survey
YLL: year of life lost
