# Supplementary material for: Development of the Impacts of Cycling Tool (ICT): A modelling study and web tool for evaluating health and environmental impacts of cycling uptake
Source: PLoS Med. 2018 Jul 31;15(7):e1002622. doi: 10.1371/journal.pmed.1002622 (PMC6067715; doi:10.1371/journal.pmed.1002622)
Supplement: S1 Text — (DOCX) [file pmed.1002622.s001.docx]

#### S1: Calculating the total weekly physical activity of individuals

Since the health benefits of extra physical activity are greatest among individuals who are otherwise doing little total physical activity ((17,41,47); see also below), we also estimated non-travel physical activity by imputing values through a matching process with similar individuals from the 2013-14 APS (Active People Survey). Background leisure time physical activity (MMETh/week) was calculated for each APS individual in two ways. For calculation of health outcomes we only included those domains of activity that correspond to the domains in the study that we used for the relative risks (22). These domains were based on self-reported sport, plus moderate to vigorous ‘DIY’ (do-it-yourself) and garden activity. Time spent walking and cycling was used for matching with the NTS values.

Physical activity energy expenditure in other sports and recreational activities was based upon APS information on participation in 420 separate activities. For each of these activities, APS records the number of days in the past four weeks on which the person participated in the activity; and the average duration of participation in that activity on each of those days. For over 95% of activities, additional information is collected regarding the intensity of participation in that physical activity. For each of the 420 activities, we looked up standard MET values using the 2011 Compendium of Physical Activities, assigning higher MET values to activities if the participant reported that it raised their breathing rate or made them out of breath or sweaty. We then converted these MET values into marginal METs and summed the MMET hours across all 420 activities.

Each individual in NTS is matched to a single individual from the Active People Survey (APS) based on age (14 bands), gender (male/female), English region, socio-economic status, ethnicity (white/non-white), walking time, and cycling in the last week. For cycling there was a good correspondence between those reporting cycling at least weekly (9.6% NTS vs 9.5% APS). For walking (due to the much higher percentage of the population reporting the behaviour) we used four groups. Band were chosen for matching to give similar proportions of people in each band (S Table 1 below), even those these corresponded to notably different walking durations.

S Table 1: Walking categories in NTS and APS

| **Category** | **NTS all walking all trips** | **APS total walking time ‘dur_walk10_all_wk’** |
| --- | --- | --- |
| 1 | 0 hours [57.4%] | < 2.5 hours [55.7%] |
| 2 | >0 hours to <2 hours [19.3%] | 2.5 hours to <5 hours [20.7%] |
| 3 | 2 hours to < 4 hours [12.1%] | 5 hours to <10 hours [13.7%] |
| 4 | 4 hours +       [10.7%] | 10 hours + [10%] |

This step matches for 99.9% of NTS individuals (137,642 out of 137,411). For the remaining 162 individuals, the match is performed using a subset of 4 of the 6 previous variables (age bands, gender, region, and walking time).

The total physical activity of each individual at baseline is then calculated as:

Total MMET = MMET_1_ (active travel in all trips, from NTS) + MME

T_2_ (non-travel activities, from APS)

The non-travel activities are assumed not to change under the scenarios.
