# Supplementary material for: Development of the Impacts of Cycling Tool (ICT): A modelling study and web tool for evaluating health and environmental impacts of cycling uptake
Source: PLoS Med. 2018 Jul 31;15(7):e1002622. doi: 10.1371/journal.pmed.1002622 (PMC6067715; doi:10.1371/journal.pmed.1002622)
Supplement: S2 Text — (DOCX) [file pmed.1002622.s002.docx]

#### 2S2: Probability of cycling a trip

S Fig 1 shows that, among cyclists, the proportion of trips of 1.5 to 2.5 miles that are cycled is around 34% in young females and 42% in young males. S Fig 1 also shows that for both males and females the probability of cycling a trip decreases with greater trip distance, but this decrease happens somewhat faster for females and for older people. In applying these distance-based probabilities, we assumed that these differences by age and gender reflect physical abilities, and we therefore retained these differences under the equity scenarios. In other words, equity scenarios equalise the probability that males and females become cyclists, but do not equalise the probability that a male versus a female cyclist cycles a particular trip of a certain distance.


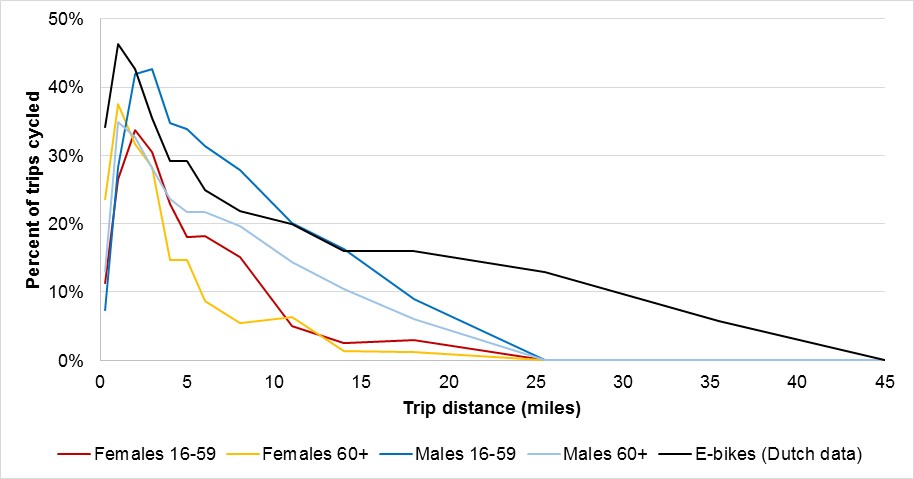


S Fig 1: Probability of a cycling a trip of a given distance among adult English cyclists in the National Travel Survey, and among adult Dutch e-bike owners

In the ‘e-bike’ versions of scenarios^[[1]](#footnote-1)^, the probabilities of cycling longer trips are generated from adults age 18-84 in the Netherlands Travel Survey (2013-14). Using these data, we defined e-bike cyclists as those who has access to an e-bike, which was 10.1% of the sample population. For these e-bike owners we calculated the probability that they cycled trips of different lengths (see S Table 2). Interestingly we found that e-bike owners did not use e-bikes for all their cycling trips but for the shortest trips also used traditional bikes – for example, e-bike owners used traditional bikes for almost a third of their cycle trips with distance under 3 miles (see S Table 2). We calculated e-bike probabilities of cycling trips of different lengths without stratifying by gender or age, under the assumption that e-bikes allow women/older people women to cycle as far as men/younger people.

Note that the probabilistic application of the distance-based probabilities means that an individual who becomes a regular cyclist (step 1) will not always have any trips switched to cycling (step 2). This could be because she did not in fact make any trips in the past week^[[2]](#footnote-2)^; because all of her actual trips were too long; or because, by chance, none of her shorter trips were switched to cycling. Thus the population can be split into four groups:

1. **Existing cyclist:** an individual who reports trips made by cycle in their travel diary, and so is a cyclist in both the baseline population and in the scenario.
2. **Regular cyclist:** an individual who is not a cyclist in the baseline population, but who is modelled as becoming a cyclist in the scenario (but may or may not actually switch any trip to cycling)
3. **New cyclist:** an individual who is not a cyclist in the baseline population, who is modelled as becoming a cyclist in the scenario, *and who switches* at least one of their trips to cycling. New cyclists are thus a subset within regular cyclists.
4. **Non-cyclist**: an individual who is not a cyclist in the baseline population, and who is not modelled as becoming a regular cyclist in the scenario.

S2 Table 1^[[3]](#footnote-3)^: Probability of Cycling a Trip of a Given Distance in the week diary among Cyclists

| **Distance (miles)** | **Female 18-59** | **Female 60-84** | **Male 18-59** | **Male 60-84** |
| --- | --- | --- | --- | --- |
| <0.5 miles | 11.3% | 23.2% | 7.4% | 13.9% |
| 0.5 to <1.5 miles | 26.5% | 37.3% | 28.3% | 34.0% |
| 1.5 to <2.5 miles | 33.8% | 30.1% | 42.0% | 33.0% |
| 2.5 to <3.5 miles | 30.4% | 28.4% | 42.7% | 28.0% |
| 3.5 to <4.5 miles | 22.9% | 14.7% | 34.8% | 23.6% |
| 4.5 to <5.5 miles | 18.1% | 14.7% | 33.9% | 21.9% |
| 5.5 to <6.5 miles | 18.1% | 8.7% | 31.4% | 21.9% |
| 6.5 to <9.5 miles | 15.1% | 5.8% | 27.9% | 18.2% |
| 9.5 to <12.5 miles | 5.0% | 5.8% | 20.2% | 14.6% |
| 12.5 to <15.5 miles | 2.7% | 1.3% | 16.3% | 10.5% |
| 15.5 to <20.5 miles | 2.7% | 1.3% | 8.9% | 6.0% |
| >20.5 miles | 0.0% | 0.0% | 0.0% | 0.0% |

S2 Table 2: Probability of cycling trips of different distance in e-bike scenario, and probability that the cycle in question is an e-bike.^[[4]](#footnote-4)^

| Distance (miles) | Prob. of cycling the trip by: | | | If the trip is cycled, what is the probability Probability that it is an e-bike  ([B]/[A+B]) |
| --- | --- | --- | --- | --- |
| **Distance (miles)** | **Traditional bike**  **[A]** | **E-bike**  **[B]** | **Any type of bike**  **[A + B]** |  |
| <0.5 | 4.7% | 5.8% | 10.5% † | 55% |
| 0.5 to <1.5 | 9.3% | 19.8% | 29.1% † | 68% |
| 1.5 to <2.5 | 9.4% | 28.3% | 37.7% † | 75% |
| 2.5 to <3.5 | 7.0% | 29.6% | 36.6% † | 81% |
| 3.5 to <4.5 | 3.1% | 25.7% | 28.8% | 89% |
| 4.5 to <5.5 | 3.1% | 25.7% | 28.8% | 89% |
| 5.5 to <6.5 | 2.4% | 22.7% | 25.1% | 90% |
| 6.5 to <9.5 | 1.6% | 20.0% | 21.5% | 93% |
| 9.5 to <12.5 | 1.1% | 19.0% | 20.1% | 95% |
| 12.5 to <15.5 | 0.8% | 15.3% | 16.1% | 95% |
| 15.5 to <20.5 | 0.8% | 15.3% | 16.1% | 95% |
| 20.5 to 30.5 | 0.0% | 12.6% | 12.6% | 100% |
| 30.5 to 40.5 | 0.0% | 5.9% | 5.9% | 100% |
| >40.5 | 0.0% | 0.0% | 0.0% | - |

S2 Table 3: Speeds for bikes (speeds for traditional bikes were calculated from the NTS, and for e-bikes from a Dutch report)

| Age band | Speed (males, mph) | Speed (females, mph) | Speed (e-bikes) |
| --- | --- | --- | --- |
| 18-29 | 11.28 | 10.46 | 11.28 (48) |
| 30-49 | 10.81 | 10.07 |  |
| 50-59 | 10.53 | 9.82 |  |
| 60-69 | 9.59 | 9.01 |  |
| 70 plus | 8.82 | 7.90 |  |

1. E-bikes come in different types. Here we are referring to Pedelec or pedal assist bikes the most common type of e-bikes in the Netherlands. To use these bikes pedalling is required at all times but different levels of electric assistance are provided. [↑](#footnote-ref-1)
2. Some people in the NTS do not have any trips. They are included in the model for estimating population averages, but their lack of travel is assumed as a constant under all scenarios [↑](#footnote-ref-2)
3. Due to small cell sizes, data was averaged for younger females cycling 4.5 to 6.5 miles, and cycling 12.5 to 20.5 miles; for older females cycling 3.5 to 5.5 miles, and cycling 6.5 to 12.5 miles;, and for older males cycling 4.5 to 6.5 miles. Among older males, we also imputed the probability of cycling trips 15.5-20.5 miles based on the probability of cycling trips 15.5-20.5 miles in younger males and the probability of cycling trips 12.5-15.5 miles in older males. [↑](#footnote-ref-3)
4. Due to small cell sizes, data was averaged for trips 3.5 to 5.5 miles, and for trips 12.5 to 20.5 miles. These data are largely taken from Dutch e-bike owners, with the exception of the four cells marked ‘†’ in the third column. In these marked cells, which all relate to trips of <3.5 miles, the values for English adults were used instead. This decision was taken because we specifically wanted to model the plausible effect of e-bikes in extending the distance that English adults were willing to cycle, rather than any effect that e-bike ownership might have on short trips. [↑](#footnote-ref-4)
