# Supplementary material for: Development of the Impacts of Cycling Tool (ICT): A modelling study and web tool for evaluating health and environmental impacts of cycling uptake
Source: PLoS Med. 2018 Jul 31;15(7):e1002622. doi: 10.1371/journal.pmed.1002622 (PMC6067715; doi:10.1371/journal.pmed.1002622)
Supplement: S3 Text — (DOCX) [file pmed.1002622.s003.docx]

#### S3: Further details of health calculations

The health impact of cycling is calculated using a Comparative Risk Assessment (CRA) with exposure data estimated at the individual level. The health impact was calculated for age group and gender specific premature mortality, including both premature deaths averted and years of life lost (YLLs)^[[1]](#footnote-1)^ taken from the Global Burden of Disease data(49,50). We estimated the relative risk on all-cause mortality for a change in non-work related physical activity for each individual using a dose-response curve (S Fig 2) from a large pooled meta-analysis of cohort studies (28) . To calculate the proportional reduction in disease burden resulting from the increase in physical activity, we calculated population impact fractions (PIFs) using the formula

**PIF** = $\frac{\sum RRb- \sum RRsc}{\sum RRb}$

Where $RRb$ is the baseline relative risk, $RRsc$ is the scenario specific relative risk, and these are summed across all individuals (i.e. treating each individual as a stratum of 1/n). For each age/gender stratum, we calculated PIFs and then multiplied each PIF by the disease burden; results were then summed over all age/gender strata. Disease burden was defined as premature deaths averted and years of life lost (YLLs), taken from the Global Burden of Disease data for England and each region in England (49,50).

**Dose response relationship**

Estimates of the impact of different amounts of physical activity on all-cause mortality were taken from a very large pooled analysis of leisure time physical activity (661 137 men and women)(22). From this study we derived a dose response curve for non-work physical activity measured as MMETh per week, see S Fig 2 below. In line with previous studies (41,47) the dose response curve is such that moving from no activity to a small amount produces the largest benefit. Given the non-linear relationship the benefits from physical observed in this study are, as expected, greater than from meta-analyses of walking or cycling alone (approximately RR 0.69 versus RR 0.83 for cycling and RR 0.91 for walking at 11.25 MMET hours per week). This does not mean that our method estimates larger health impacts than the walking and cycling specific risks as our population including will be starting further down the dose response curve than would a population for which only active travel is included.


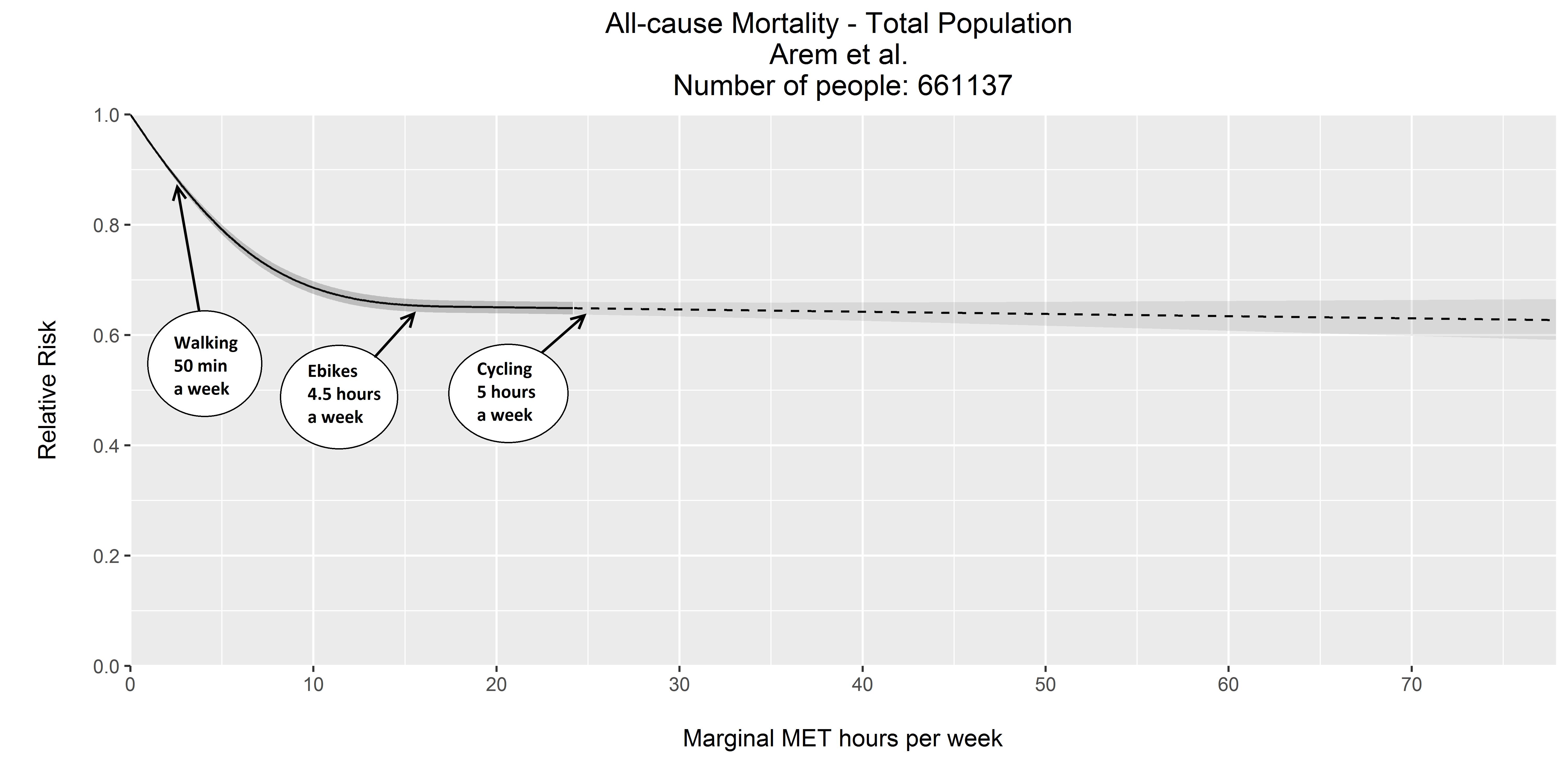


S3 Fig 2: Dose response curve giving the relative risks for all-cause mortality for different physical activity levels (MMETh per week)

##### Population Impact Fraction

Population Impact Fraction (PIF) is the proportional reduction in population mortality (or disease burden), following a change in exposure to a risk factor. PIF can be represented in the following way.

PIF =$\frac{attribublecases}{totalcases}$

In other words,

PIF = $\frac{factualcases-counterfactualcases}{factualcases}$

PIF = $\frac{\sum RRb- \sum RRsc}{\sum RRb}$

where $RRb$is the relative risk at baseline, and $RRsc$is the relative risk for the scenario. In our case the exposure is MMETh per week for each individual. Using the dose response curve illustrated in S Fig 2 previously we can estimate the relative risk (RR) for a change in exposure for each individual. The resulting PIF can then be applied to age group and gender specific disease burden data.

##### Burden of Premature Mortality

Years of life lost and premature deaths are taken from the Global Burden of Disease data for the UK. These data are presented by gender and for five year age categories. The method used could be extended for morbidity data (years of healthy life lost due to illness or disability) and disease specific mortality.

To summarise six steps are needed to complete the health calculations:

1. Calculate the individual level of physical activity (MMETh per week).
2. Calculate Relative Risks, for the each individual based on total MMETh (22).
3. Apply CRA formula – treating each individual as a strata of 1/n

PIF = $\frac{\sum RRb- \sum RRsc}{\sum RRb}$

, where $RRb$is the baseline relative risk and $RRsc$ is the scenario specific relative risk.

1. PIFs are calculated separately for each age/gender strata
2. PIF * Disease burden for each age/gender strata
   1. Global Burden of Diseases (GBD) data UK all-cause mortality
3. Sum results over age/gender strata

1. YLLs refer to the burden of premature mortality compared with a full life expectancy for that age. The burden is per ‘accounting year’, that is it refers to the postponing of deaths that would occur in one year but the extra years then lived will stretch out into the future until the full life expectancy is reached. [↑](#footnote-ref-1)
