# Supplementary material for: Development of the Impacts of Cycling Tool (ICT): A modelling study and web tool for evaluating health and environmental impacts of cycling uptake
Source: PLoS Med. 2018 Jul 31;15(7):e1002622. doi: 10.1371/journal.pmed.1002622 (PMC6067715; doi:10.1371/journal.pmed.1002622)
Supplement: S4 Text — YLL, year of life lost. (DOCX) [file pmed.1002622.s004.docx]

|  | **Mode** | | | | | | | |
| --- | --- | --- | --- | --- | --- | --- | --- | --- |
| **Scenario: 25 %** | **walk** | **bike** | **ebike** | **car driver** | **car passenger** | **bus** | **train** | **other** |
| ebike=off, equity=off | 20.2% | 6.3% | 0.0% | 49.9% | 13.9% | 5.3% | 2.8% | 1.6% |
| ebike=on, equity=off | 20.1% | 2.6% | 3.9% | 49.8% | 13.8% | 5.3% | 2.8% | 1.6% |
| ebike=off, equity=on | 20.1% | 6.0% | 0.0% | 50.3% | 13.8% | 5.3% | 2.8% | 1.7% |
| ebike=on, equity=on | 20.1% | 2.6% | 3.9% | 49.9% | 13.7% | 5.3% | 2.8% | 1.6% |

S4 Table 1: Mode share for all four possible combinations of 25% scenario

| Scenario: 25% | 18-39 | | 40-59 | | 60-79 | |
| --- | --- | --- | --- | --- | --- | --- |
|  | m | f | m | f | m | f |
| ebike=off, equity=off | 2.7% | 1.1% | 3.8% | 1.4% | 2.0% | 0.6% |
| ebike=on, equity=off | 2.5% | 1.3% | 3.4% | 1.6% | 2.0% | 0.7% |
| ebike=off, equity=on | 1.6% | 1.6% | 2.3% | 1.9% | 2.3% | 1.6% |
| ebike=on, equity=on | 1.5% | 1.8% | 2.1% | 2.1% | 2.2% | 2.2% |

S4 Table 2: YLL reductions for males and females, for all four possible combinations of 25% scenario
