# Supplementary figures and images for: Development of the Impacts of Cycling Tool (ICT): A modelling study and web tool for evaluating health and environmental impacts of cycling uptake
Source: PLoS Med. 2018 Jul 31;15(7):e1002622. doi: 10.1371/journal.pmed.1002622 (PMC6067715; doi:10.1371/journal.pmed.1002622)

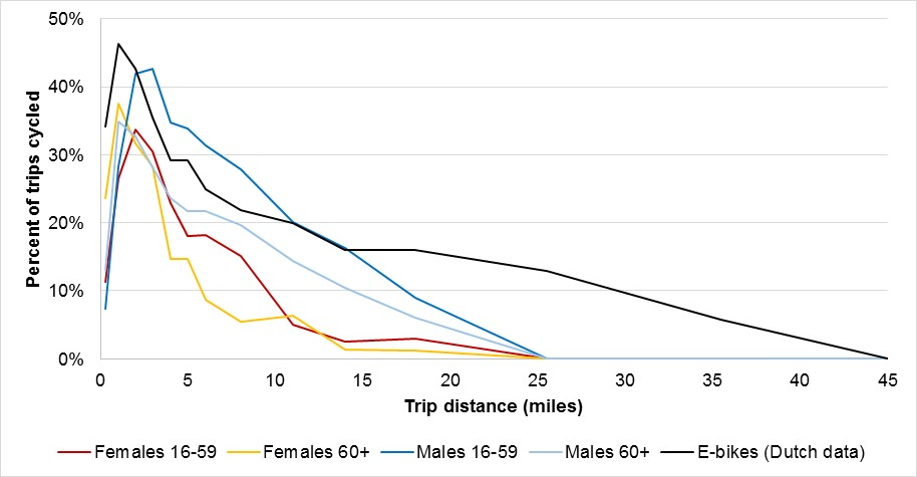

Supplement: S1 Fig — (PNG) [file pmed.1002622.s007.png]

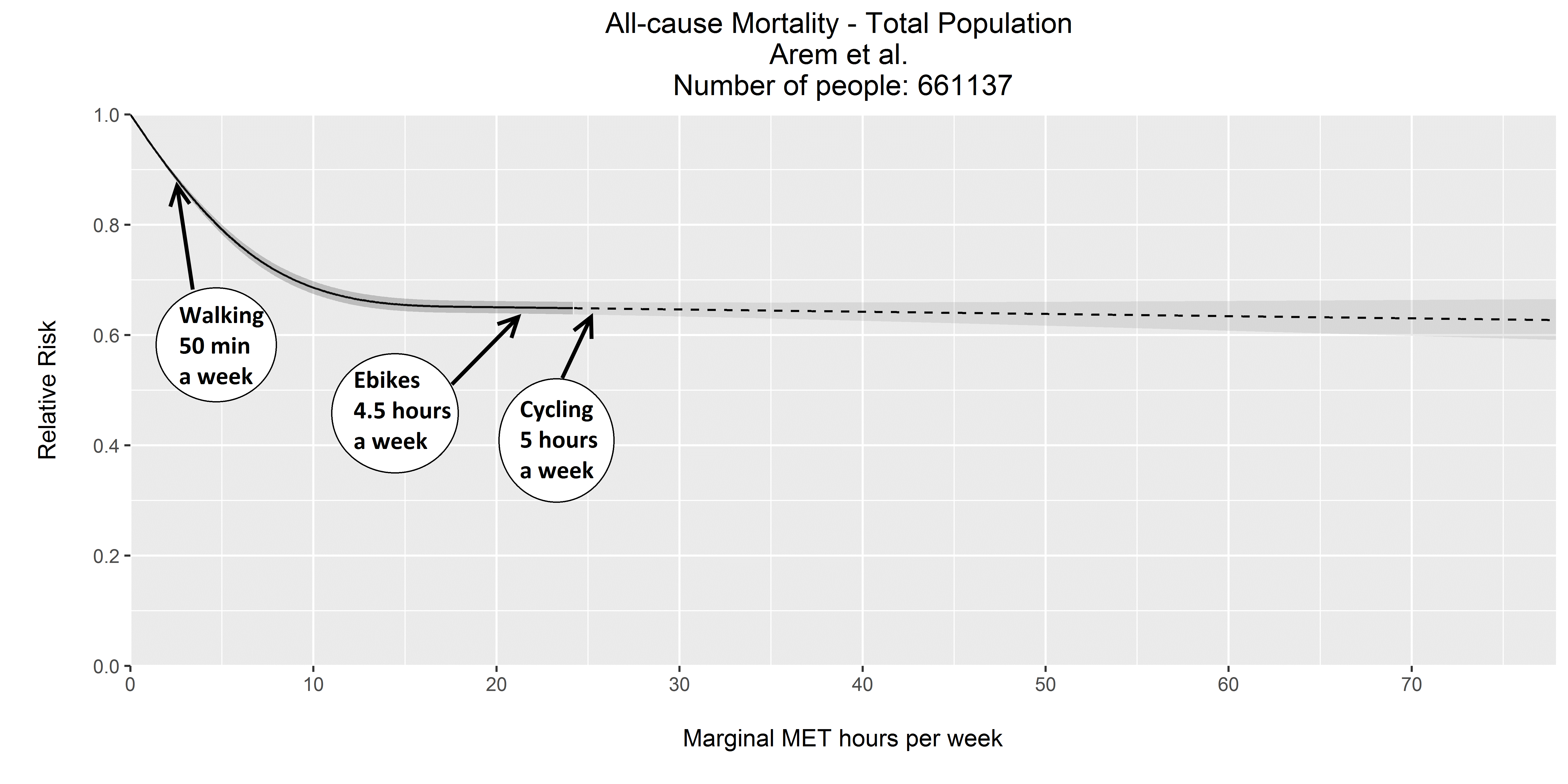

Supplement: S2 Fig — (PNG) [file pmed.1002622.s008.png]

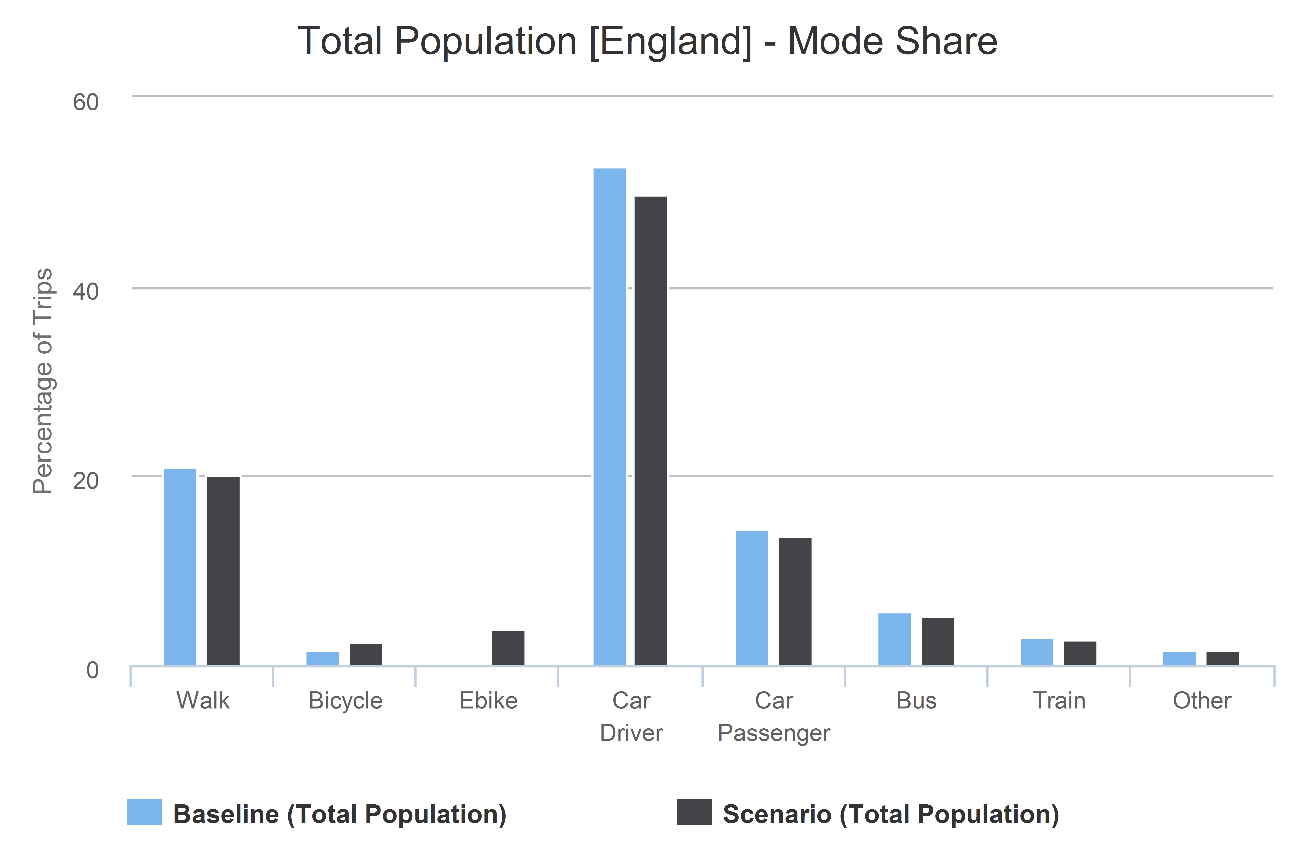

Supplement: S3 Fig — (PNG) [file pmed.1002622.s009.png]

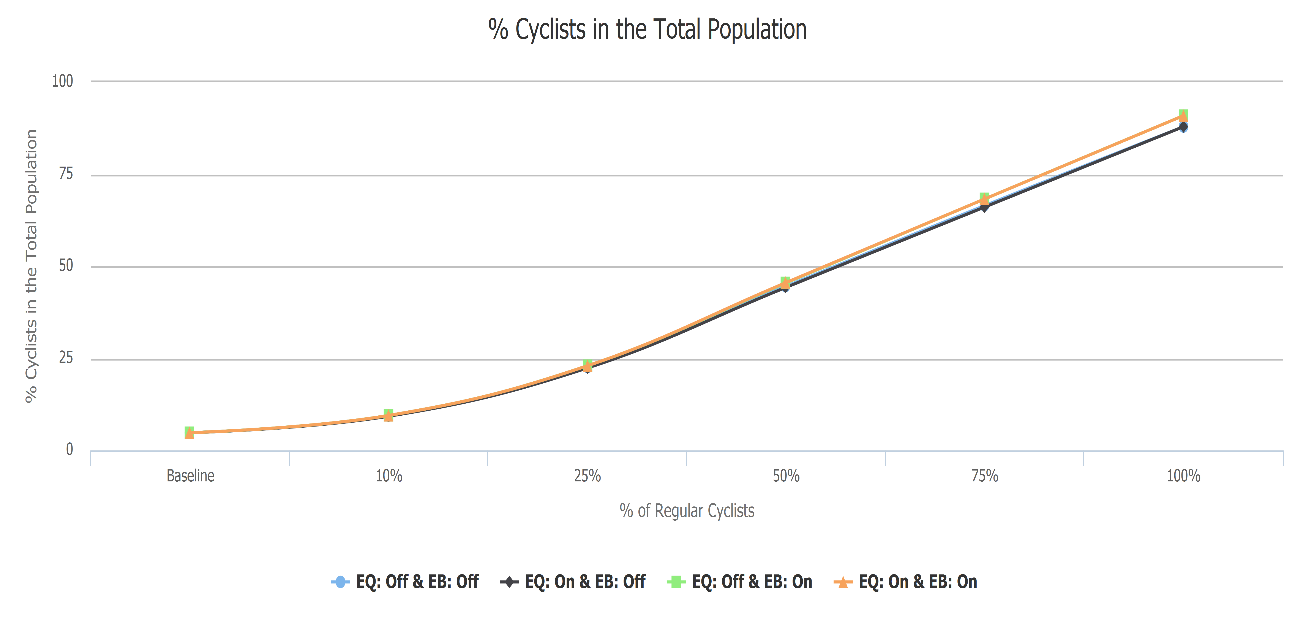

Supplement: S4 Fig — (PNG) [file pmed.1002622.s010.png]

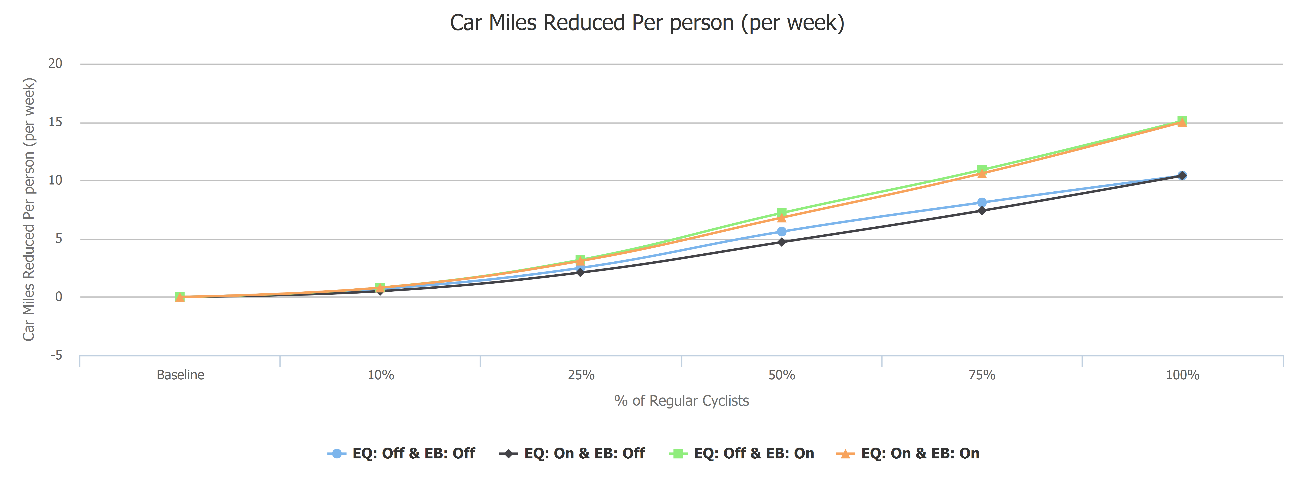

Supplement: S5 Fig — (PNG) [file pmed.1002622.s011.png]

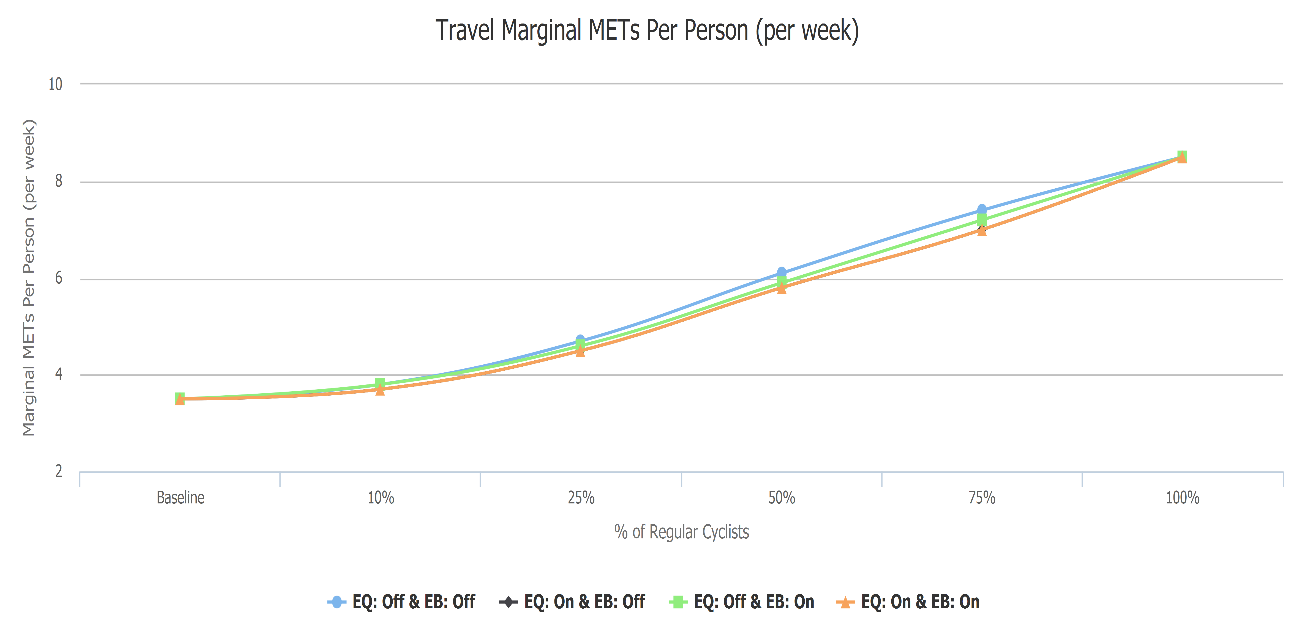

Supplement: S6 Fig — EB, e-bikes scenario; EQ, equity scenario; MET, Metabolic Equivalent Task. (PNG) [file pmed.1002622.s012.png]

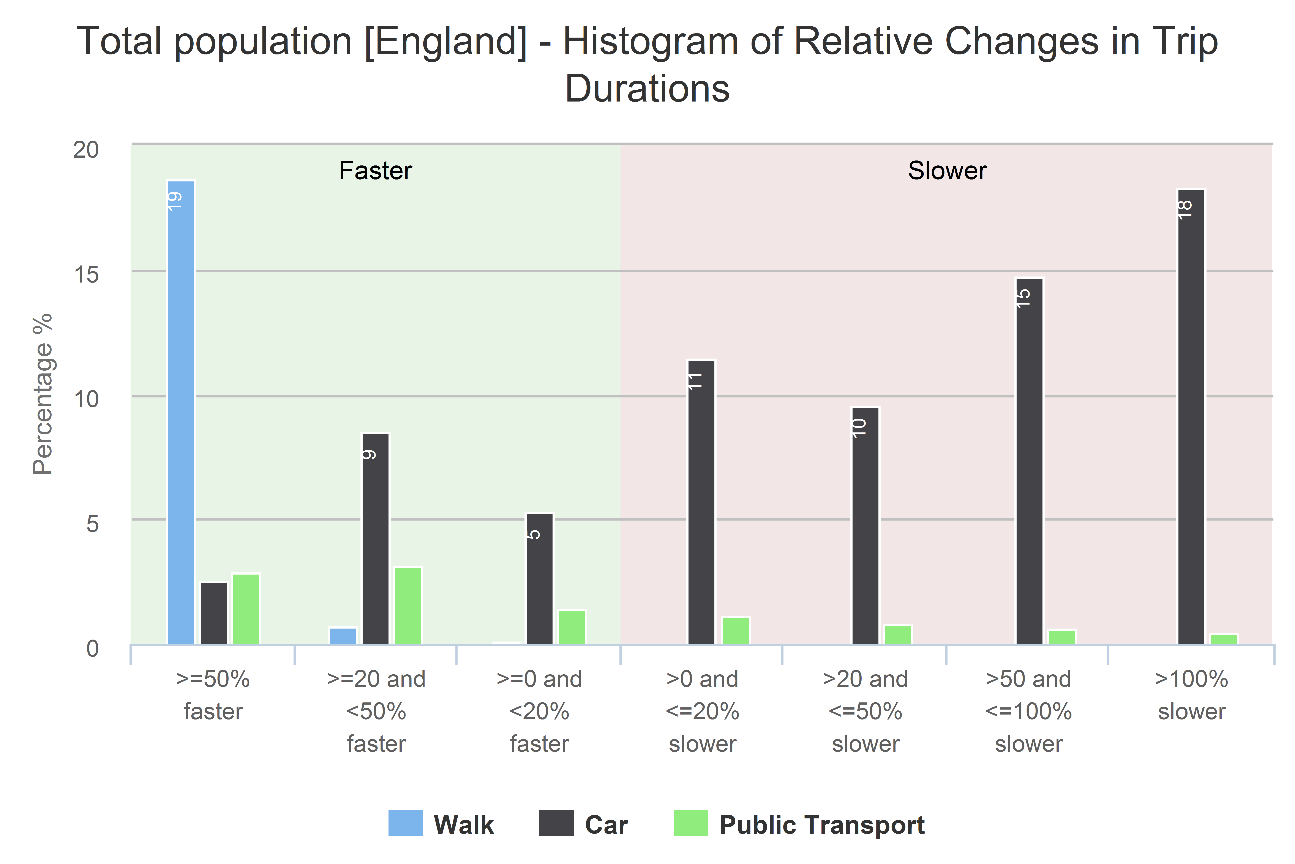

Supplement: S7 Fig — Scenario regular cyclists 100%, equity on, e-bikes off. (PNG) [file pmed.1002622.s013.png]
